# Strategic targeting of Cas9 nickase expands tandem gene arrays

**DOI:** 10.1016/j.xgen.2025.100811

**Published:** 2025-03-20

**Authors:** Hiroaki Takesue, Satoshi Okada, Goro Doi, Yuki Sugiyama, Emiko Kusumoto, Takashi Ito

**Affiliations:** 1 Department of Biochemistry, Kyushu University Graduate School of Medical Sciences, Fukuoka 812-8582, Japan

**Keywords:** nCas9, replication fork, structural variation, genome editing

## Abstract

Expanding tandem gene arrays facilitates adaptation through dosage effects and gene family formation via sequence diversification. However, experimental induction of such expansions remains challenging. Here, we introduce a method termed break-induced replication (BIR)-mediated tandem repeat expansion (BITREx) to address this challenge. BITREx places Cas9 nickase adjacent to a tandem gene array to break the replication fork that has just replicated the array, forming a single-ended double-strand break. This break is subsequently end-resected to become single stranded. Since there is no repeat unit downstream of the break, the single-stranded DNA often invades an upstream unit to initiate ectopic BIR, resulting in array expansion. BITREx has successfully expanded gene arrays in budding yeast, with the *CUP1* array reaching ∼1 Mb. Furthermore, appropriate splint DNAs allow BITREx to generate tandem arrays *de novo* from single-copy genes. We have also demonstrated BITREx in mammalian cells. Therefore, BITREx will find various unique applications in genome engineering.

## Highlights

• BITREx uses Cas9 nickase to expand tandem gene arrays by break-induced replication
• BITREx can generate megabase-sized gene arrays in budding yeast
• Splint DNA allows BITREx to form a tandem array *de novo* from a single-copy gene
• BITREx is applicable to mammalian cells

## Graphical abstract

**Figure:**
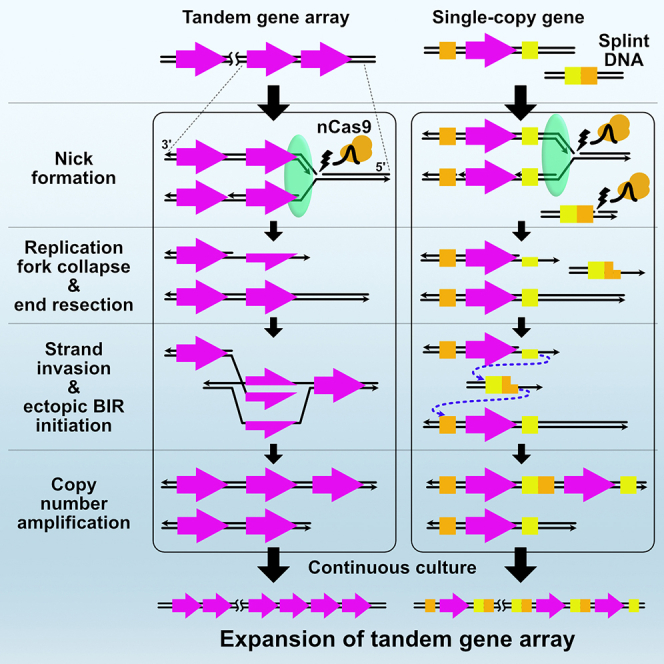

## Teaser

Takesue et al. introduce BITREx, a novel genome editing method that strategically directs a single Cas9 nickase adjacent to a target tandem gene array, enabling its efficient expansion. By leveraging replication fork breakage, BITREx drives copy-number amplification, offering a unique approach to genome manipulation.

## Introduction

Gene duplication plays a critical role in adaptation and evolution, as has become increasingly apparent with the advent of comparative and personal genomics. Two major mechanisms of gene duplication are retrotransposition and non-allelic homologous recombination.^1^ The latter mechanism initially generates a tandemly duplicated pair of the gene, and this configuration often invites additional recombination events to form a tandem gene array. Expansion and contraction of such arrays result in copy-number variation (CNV) of the gene.

An immediate effect of expanding tandem gene arrays would be dosage effects (Figure S1A). Increased gene dosage often contributes to adaptation to environmental changes. For example, the copy number of an amylase gene, *AMY2B*, shows a notable difference between domestic dogs and wolves, both of which belong to the same species, *Canis lupus*: domestic dogs have more copies than wolves.^2^ This is probably because domestic dogs have adapted to the carbohydrate-rich diet provided by humans, whereas wolves have maintained the ancestral copy number of *AMY2B* without finding any benefit from its high dosage in the wild.

Similarly, the budding yeast *Saccharomyces cerevisiae* shows CNV of the *CUP1* gene, which encodes a copper metallothionein. Different strains have *CUP1* arrays with varying numbers of repeat units, resulting in different levels of copper resistance. Interestingly, the boundaries of the repeat units composing the *CUP1* arrays vary between strains, suggesting that the initial duplication events from a single-copy *CUP1* gene to a two-unit *CUP1* array occurred independently in their ancestors.^3^ Thus, the formation of the *CUP1* array is an example of convergent evolution. It is considered to have occurred during domestication because *CUP1* is a single-copy gene in many wild isolates of *S. cerevisiae* and in *S. paradoxus*, a non-domesticated cousin of baker’s yeast.^4^ These yeasts are unlikely to have been exposed to high concentrations of copper in the wild, and thus would not have seen any fitness benefit from increased *CUP1* dosage. Expanding tandem gene arrays is a powerful strategy for rapid adaptation to environmental change. Indeed, clones with expanded *CUP1* arrays can be easily obtained in the laboratory by growing yeast cells in the presence of high copper concentrations.^5^

The expansion of tandem gene arrays can also have long-term effects, including sequence diversification that generates paralogs (Figure S1A). This process allows the original tandem gene array to evolve into a genomic locus encoding members of a multigene family. A paradigmatic example of such a locus is the human β-globin locus, which is composed of five genes and one pseudogene. This arrangement allows for the developmental stage-dependent production of three different isoforms—embryonic, fetal, and adult hemoglobins—enabling adaptation to change in oxygen concentration. Extreme examples of gene families with tandemly arrayed paralogs include those for the olfactory receptor (OR), immunoglobulin, and cytochrome P450. For instance, the human and elephant genomes contain ∼400 and ∼2,000 OR genes, respectively, with nearly as many pseudogenes.^6^ While pseudogenization appears to be an inevitable consequence of the functional differentiation of duplicated/multiplicated genes, certain pseudogenes participate in gene conversion, contributing to the immune system, and others play biological roles by generating functional non-coding RNAs.^7^ Tandem gene amplification followed by sequence diversification is therefore a fundamental strategy in evolution by gene duplication.

The recent advent of genome editing has made it possible to manipulate tandem gene arrays. Cleaving the repeat units of a tandem gene array easily induces its contraction through single-strand annealing (SSA) or non-homologous end joining (NHEJ). In contrast, there is currently no method to expand a tandem gene array. Developing such a method could induce dosage effects and serve as a potential strategy for increasing the yield of useful gene products. It could also provide a basis for generating multigene families, serving as a unique tool for experimental evolution to enhance the potential of cells. But how can we expand a tandem gene array?

In this context, it is interesting to note our previous finding that targeting the catalytically inactive variant of Cas9 (dCas9) to the *CUP1* array induces its contraction in the majority of cells and expansion in a minority of cells.^8^ Mechanistically, dCas9 interferes with replication fork progression, and some of the stalled forks likely break, leading to recombinational repair events that inevitably include non-allelic recombination, resulting in *CUP1* CNV. On the other hand, a single-molecule observation study revealed that replisome disassembles upon collision with Cas9 nickase (nCas9).^9^ Based on our considerations of the potential mechanism for dCas9-induced *CUP1* array expansion and the nCas9-induced replisome disassembly observed by others, we conceived the idea of repurposing break-induced replication (BIR)^10^^,^^11^—a mechanism to repair single-ended double-strand breaks (seDSBs) generated upon replication fork breakage—to expand tandem gene arrays by strategically targeting nCas9.

## Design

Breakage of a replication fork results in the formation of a seDSB. Subsequent end-resection of the seDSB converts it into a 3′-protruding single-stranded DNA (ssDNA). The ssDNA typically invades the sister chromatid at its allelic position and initiates displacement synthesis, known as BIR (Figure S1B), which continues until it encounters a converging replication fork.^10^^,^^11^^,^^12^ When a replication fork collapses within a tandem gene array, the cell can initiate BIR either orthotopically (i.e., at the allelic position) or ectopically (i.e., at non-allelic positions) (Figure S1C). While orthotopic BIR preserves the tandem gene array, ectopic or out-of-register BIR induces copy-number alterations (CNA) of repeat units. If the ssDNA enters a repeat unit downstream or upstream of the seDSB with respect to the direction of replication fork progression, subsequent BIR will decrease or increase the copy number of the repeat units, resulting in contraction or expansion of the array, respectively.

**Figure 1:**
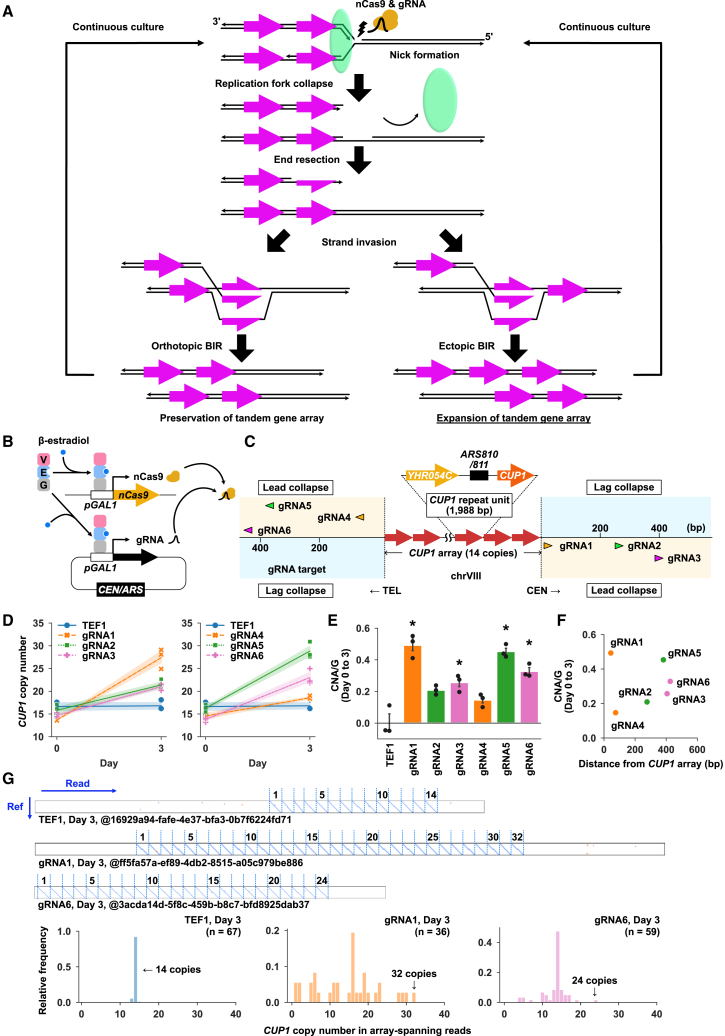
Proof of concept for BITREx by nCas9-induced *CUP1* array expansion (A) Principle of BITREx. (B) GEV-based system for co-induction of nCas9 and gRNA. (C) *CUP1* array and target sites of effective gRNAs (gRNA1-gRNA6). A rightward or leftward arrowhead indicates that the gRNA sequence is designed for the top or bottom strand, respectively, with its protospacer adjacent motif (PAM). Consequently, the nCas9-gRNA complex nicks the bottom or top strand, which serves as a template for leading strand synthesis initiated from the *ARS810/811* in the *CUP1* repeat unit (lead collapse). (D) nCas9-induced CNA of *CUP1*. Expression of nCas9 and gRNAs were induced by the addition of 10 nM β-estradiol on day 0. Population average *CUP1* copy number was determined by qPCR on days 0 and 3. Each point in the line plot indicates the average *CUP1* copy number (*n* = 3 biological replicates), with shading around each line indicating the standard deviation (SD). (E) nCas9-induced CNA/G of *CUP1*. CNA was divided by the number of cell divisions estimated from the increase in the optical density of the culture. Error bar represents SD (*n* = 3 biological replicates). ∗*p* < 0.05 (Student’s t test). (F) Plot of the CNA/G value versus the distance from the *CUP1* array to each gRNA target sequence. (G) Nanopore sequencing of *CUP1* arrays. Upper panel, representative dot plots comparing nanopore reads to the *CUP1RU* reference sequence. Lower panel, distribution of *CUP1RU* copy numbers in the specified number of nanopore reads spanning the entire array.

We hypothesized that, if a replication fork collapses just before completing the replication of a tandem gene array to generate a seDSB within the terminal repeat unit, then the ssDNA derived from the seDSB must invade either the allelic repeat unit or an upstream repeat unit, as there is no downstream repeat unit (Figure 1A). In other words, the array loses the opportunity to contract and thus either remains unchanged or expands. Although we can use nCas9 to induce a seDSB in a replication-dependent manner, we cannot target it selectively to the terminal unit because all repeat units share an identical sequence. However, BIR can also occur, albeit with reduced efficiency, when the invading ssDNA has a 3′-tail sequence that is not homologous to the donor sequence.^13^ Therefore, we hypothesized that targeting nCas9 to the flanking site of a tandem gene array could induce its expansion. We termed this strategy BIR-mediated tandem repeat expansion (BITREx). We first investigated the feasibility of BITREx using the budding yeast *S. cerevisiae* as a model system.

## Results

### Proof of concept for BITREx by nCas9-induced *CUP1* array expansion

We constructed a strain in which β-estradiol induces the expression of Cas9^D10A^, the nCas9 that selectively cleaves the target strand hybridized with guide RNA (gRNA). The induction is mediated by the artificial transcription factor GEV, which consists of the Gal4 DNA-binding domain, estrogen receptor, and VP16 transcriptional activation domain.^14^ Upon binding to β-estradiol, the GEV migrates to the nucleus and activates *GAL1* promoters to induce the expression of Cas9^D10A^ from the genome and its gRNA from a plasmid (Figure 1B). The *CUP1* array is composed of tandem iteration of a ∼2.0-kb DNA segment that contains the *CUP1* gene and the *ARS810/811* origin of replication (referred to as the *CUP1* repeat unit or *CUP1RU*) on chromosome VIII.^3^ It consists mainly of 14 repeat units in the parental strain used in this study (14×CUP1RU, Figures S2A and S2B). We constructed a series of strains that expresses Cas9^D10A^ targeted to the upstream- and downstream-flanking sites of *CUP1* array (Figure 1C) and a control site in the *TEF1* locus on chromosome XVI.

We grew these strains by daily dilution of the culture with fresh medium, extracted genomic DNA before and 3 days after β-estradiol addition, and measured the population average *CUP1* copy number using qPCR (Figure 1D). There was no change in the *CUP1* copy number in the control strain (Figure 1D). In contrast, an increase in the *CUP1* copy number was evident in 6 out of the 24 strains with Cas9^D10A^ targeted to the flanking regions of the *CUP1* array (Figures 1D, S2C, and S2D). To evaluate the performance of each gRNA, we calculated CNA per generation (CNA/G) (Figure 1E). The 6 effective gRNAs (gRNA1–gRNA6) showed significantly higher CNA/G values than the other 18 gRNAs (gRNAs1–gRNAs18) and the control *TEF1* gRNA (Figures 1E and S2D). Although the CNA/G values varied among the 6 gRNAs, they did not correlate with the distance from the *CUP1* array to the gRNA target sites (Figure 1F). Notably, all 6 gRNAs direct Cas9^D10A^ to nick the template DNA strands for leading strand synthesis initiated by the replication origin *ARS810/811* in the *CUP1RU* (lead collapse). A series of experiments using a strain with an inverted gRNA1 target site (Figures S2E–S2G) and an improved Cas9^H840A^ variant^15^ to nick the strand opposite to Cas9^D10A^ (Figures S3A–S3C) confirmed the hypothesized requirement for lead collapse in BITREx and aligned with previous reports demonstrating the superiority of Cas9^D10A^ over Cas9^H840A^.^16^^,^^17^ Accordingly, we used Cas9^D10A^ throughout this study, referring to it hereafter as nCas9 for brevity.

Next, we performed the ligation-based nanopore whole-genome sequencing of DNA extracted from the strains after 3 days of BITREx induction. We selected reads containing both 5′- and 3′-flanking regions of the *CUP1* array and generated dot plots between these reads and the *CUP1RU* reference sequence. The number of diagonal lines in the dot plot indicates the number of repeat units comprising the array (Figure 1G). These analyses identified arrays consisting of ∼14, up to 32, and up to 24 copies of *CUP1RU* from strains with *TEF1* gRNA, gRNA1, and gRNA6, respectively (Figure 1G). Note that the apparent discrepancy between the nanopore-based and qPCR-based estimates of *CUP1* copy number arises because the former used only reads that span the entire array, resulting in longer arrays being included less frequently, whereas the latter used all arrays evenly, regardless of their lengths. Intriguingly, nanopore sequencing also revealed the presence of contracted arrays, even though the population average *CUP1* copy number increased (Figures 1G, S4A, and S4B). Additional nanopore read analyses revealed no detectable levels of translocation involving the *CUP1* locus, ectopic integration of circular DNAs excised from the *CUP1* array, or aneuploidy affecting chromosome VIII (Figures S4C and S4D).

We also examined the effects of mutating genes for essential BIR factors (Rad51, Pol32, and Pif1^18^) and a BIR inhibitor (Rtt109^19^) on the nCas9-induced *CUP1* array expansion. The expansion was inhibited in mutants of the essential factors and accelerated in the mutant of the inhibitor (Figures S5A–S5D), confirming that BIR mediates BITREx, as originally designed.

Taken together, targeting nCas9 adjacent to the *CUP1* array induced its expansion *in situ*, proving the principle of BITREx. Notably, in contrast to copper-induced expansion,^5^ BITREx expands the *CUP1* array without any selection pressure; it is so efficient that the average copy number of *CUP1RU* increases even in the unselected population.

### Long-term BITREx to generate megabase-sized *CUP1* arrays

**Figure 2:**
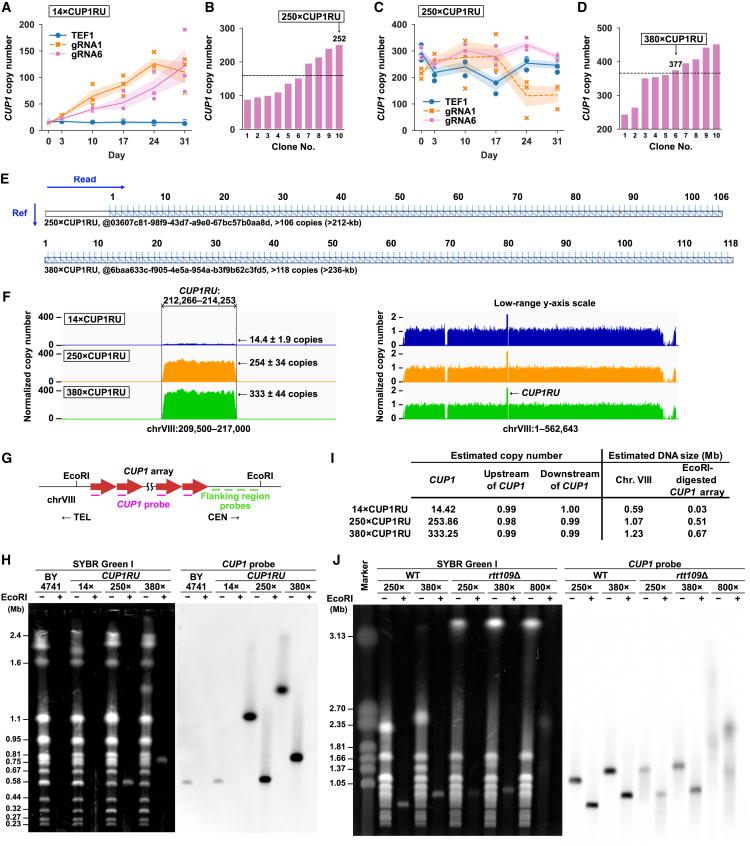
Generation of Mb-sized *CUP1* arrays by long-term BITREx (A) CNA of *CUP1* over the 31-day BITREx period. Shading, SD (*n* = 3 biological replicates). (B) *CUP1* copy number of 10 randomly picked clones on day 31 of BITREx using gRNA6. The dashed line indicates the average copy number, which is 160. (C) CNA of *CUP1* over the second 31-day BITREx period. Shading, SD (*n* = 3 biological replicates). (D) *CUP1* copy number of 10 randomly picked clones on day 31 of the second cycle of BITREx using gRNA6. The dashed line indicates the average copy number, which is 366. (E) Representative dot plots comparing nanopore reads from the 250×CUP1RU and 380×CUP1RU strains to the *CUP1RU* reference sequence. (F) Normalized read counts in Illumina sequencing. The left panel focuses on the region around *CUP1RU*, while the right panel shows the entire chromosome with a low-range y axis scale. Read counts were normalized to the average counts of genomic regions excluding rRNA, *CUP1RU*, Ty elements, and mitochondrial DNA. Note that, while the sacCer3 reference genome sequence contains two copies of *CUP1RU*, the second copy is masked with “N” prior to mapping. Consequently, the normalized read count directly reflects the *CUP1RU* copy number. The gaps in read counts on the left arm and adjacent to *CUP1RU* are due to the presence of a Ty4 element and the aforementioned masking, respectively. The dip on the right arm results from the presence of a Ty1 element and the segmental duplication between chromosomes VIII and I. (G) Strategy of Southern blot hybridization. (H) PFGE analysis of *CUP1* arrays expanded by long-term BITREx. Left, SYBR Green I stain; right, blot hybridized with the *CUP1* probe. (I) Deep sequencing-based estimates of the sizes of chromosome VIII and the EcoRI restriction fragment containing the *CUP1* array. (J) PFGE analysis of *CUP1* arrays expanded in wild-type and *rtt109*Δ cells by long-term BITREx. Left, SYBR Green I stain; right, blot hybridized with the *CUP1* probe. The most slowly migrating bands are chromosome XII, which harbors the rDNA array known to expand remarkably in *rtt109*Δ cells.^20^

Theoretically, BITREx occurs every cell cycle to continuously extend the target gene array. To test this possibility, we performed a 31-day continuous BITREx experiment by diluting the yeast cell culture every 3 days for inoculation into fresh medium. Starting from the wild-type strain with 14 copies of *CUP1RU* (14×CUP1RU), long-term BITREX using gRNA1 or gRNA6, but not *TEF1* gRNA, increased the *CUP1* copy number (Figure 2A). From the 10 clones randomly isolated from the gRNA6-expressing cell population with an estimated copy number of 160 (Figure 2B), we selected one clone estimated to carry 252 units (250×CUP1RU) for further experiments. Following the curing of the gRNA6-expressing plasmid from this clone, we transformed the cells with the *TEF1* gRNA-, gRNA1-, or gRNA6-expressing plasmid and subjected the obtained transformants for another cycle of 31-day BITREx. Intriguingly, the copy number appeared to reach a plateau (∼300) during the second cycle with gRNA6 (Figure 2C). We selected a strain estimated to carry 377 units (380×CUP1RU) from the resulting cell population for subsequent experiments (Figure 2D). In the gRNA1-expressing strain, the copy number initially appeared to plateau but subsequently declined (Figure 2C). Prolonged nicking over time can sometimes induce mutations at the target sites (e.g., Figure S6A), potentially enabling clones with defective nicking to dominate the population. However, no mutations were detected at the gRNA1 target site in these cell populations (Figure 2C), leaving the cause of the decline unexplained. In the *TEF1* gRNA-expressing control strain, the copy number fluctuated substantially but was largely maintained without showing a consistent decline (Figure 2C).

We performed nanopore sequencing to detect the expanded *CUP1* array in the 250×CUP1RU and 380×CUP1RU strains. Among the reads obtained are those containing more than 106 (>212 kb) and 118 (>236 kb) copies of *CUP1RU* (Figure 2E). However, we failed to obtain such reads that span the full length of the extended array, which must be longer than ∼500 and ∼760 kb for 250×CUP1RU and 380×CUP1RU, respectively. Nevertheless, the normalized read counts of *CUP1RU* in Illumina sequencing were 254 ± 34 and 333 ± 44 in the 250×CUP1RU and 380×CUP1RU strains, respectively, while those of non-*CUP1RU* regions on chromosome VIII were equal to one (i.e., genome-wide average) (Figure 2F). We also confirmed that non-*CUP1RU* portions of *CUP1RU*-containing nanopore reads were derived exclusively from the *CUP1* locus on chromosome VIII (Figure S6B). These results suggested that BITREx can expand the *CUP1* array *in situ* to a sub-Mb size with no gross rearrangement.

To visualize the entire chromosome VIII and the *CUP1* array in these strains, we performed pulsed-field gel electrophoresis (PFGE) followed by Southern blot hybridization using probes derived from either *CUP1RU* or its flanking region (Figure 2G). Both probes hybridized to 0.6-, 1.1-, and 1.4-Mb bands in the intact genomic DNAs derived from the 14×CUP1RU, 250×CUP1RU, and 380×CUP1RU strains, respectively (Figures 2H and S6C). We also examined EcoRI-digested genomic DNA. Since *CUP1RU* has no EcoRI site, the extended arrays appeared as 0.5- and 0.8-Mb bands in the 250×CUP1RU and 380×CUP1RU strains, respectively (Figures 2H and S6C). These sizes were consistent with those estimated from the Illumina deep sequencing (Figure 2I). We did not detect any aberrant bands indicating translocations or other chromosomal alterations.

To further explore the potential of BITREx in *CUP1* array expansion, we performed a long-term culture experiment using the *rtt109*Δ strain, as it remarkably accelerated the array expansion (Figure S5C). In the absence of Rtt109, BITREx for 31 days increased the average *CUP1* copy number to ∼500, resulting in generation of arrays exceeding 1 Mb in length (Figure S6D). We examined individual colonies by qPCR and obtained strains with estimated copy numbers of 274 (*rtt109*Δ 250×CUP1RU), 387 (*rtt109*Δ 380×CUP1RU), and 819 (*rtt109*Δ 800×CUP1RU) for further experiments (Figure S6D). After 3 days of culture without estradiol, the first two strains exhibited only a modest decrease in the *CUP1* copy number as did the wild-type strains with similar copy numbers, while the third strain showed a significant decrease from ∼700 to ∼350 copies, suggesting an intrinsic instability of extremely long *CUP1* array (Figure S6E). The normalized Illumina read counts in the *CUP1RU* increased significantly, whereas those in the non-*CUP1RU* regions on chromosome VIII did not show any detectable CNAs, ruling out the possibility of aneuploidy (Figure S6F). Non-*CUP1RU* portions of *CUP1RU*-containing nanopore reads showed no evidence of gross rearrangements (Figure S6G). Southern blot hybridization confirmed the elongation of the *CUP1* array to over 1 Mb in the *rtt109*Δ 800×CUP1RU strain despite its slow growth (Figures 2J and S6H). However, the hybridization bands in *rtt109*Δ strains appeared fuzzier than those in the wild-type strain, suggesting that *rtt109*Δ cells likely harbor more heterogeneous *CUP1* arrays than their wild-type counterparts. In addition, hyper-amplification of the rDNA array in *rtt109*Δ cells^20^ resulted in a marked delay in the migration of chromosome XII (Figures 2J and S6H).

Taken together, BITREx can expand the *CUP1* array to Mb size, especially in the absence of Rtt109. To maintain the extremely long arrays, BITREx likely needs to be continuously induced.

### Epigenetic modulation of BITREx

**Figure 3:**
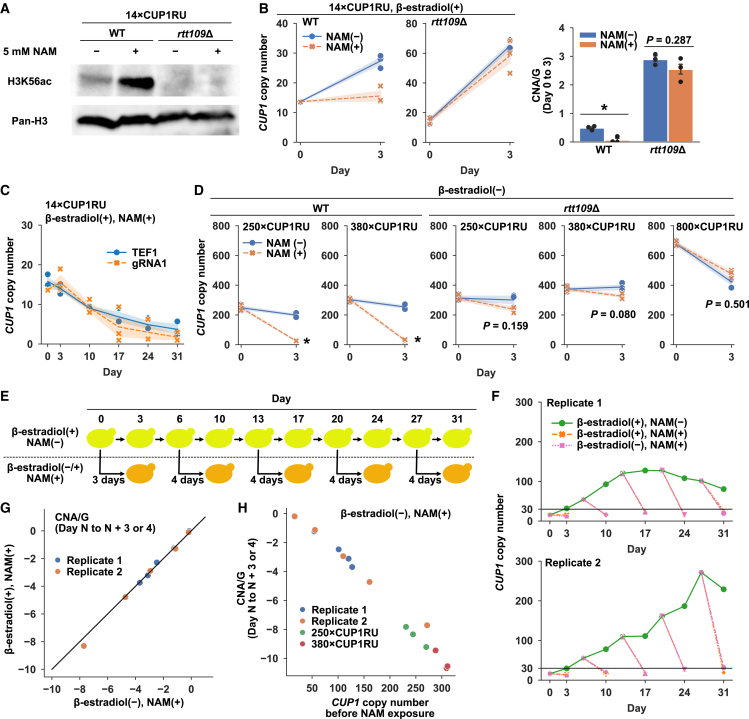
Epigenetic modulation of BITREx (A) Effects of NAM on H3K56ac. The wild-type and *rtt109*Δ cells were grown in the absence and presence of 5 mM NAM for 3 days and subjected to immunoblot analysis of H3K56ac and total histone H3. (B) Effects of NAM on BITREx of the *CUP1* array using gRNA1. Shading and error bar, SD (*n* = 3 biological replicates). ∗*p* < 0.05 (Student’s t test). (C) Effects of long-term NAM exposure on the normal *CUP1* array in the wild-type strain (14×CUP1RU) with *TEF1* gRNA and gRNA1. Shading, SD (*n* = 3 biological replicates). (D) Effects of short-term NAM exposure on the BITREx-extended *CUP1* arrays in the presence and absence of Rtt109. Note that BITREx was not induced during the NAM exposure. Shading, SD (*n* = 3 biological replicates). ∗*p* < 0.05 (Student’s t test). (E) Experimental strategy to examine the effect of initial *CUP1* copy number on NAM-induced array contraction. (F) NAM-induced contraction of variably extended *CUP1* arrays. Line plots indicate the actual data following the strategy depicted in (E) (*n* = 2 biological replicates). BITREx was induced in the wild-type strain using gRNA1. The horizontal lines indicate 30 copies. (G) Effect of BITREx on NAM-induced contraction of extended *CUP1* arrays. The CNA/G values are compared between the absence (x axis) and presence (y axis) of BITREx induction. The diagonal line indicates y = x. (H) Plot of CNA/G versus initial *CUP1* copy number. Data from (F) and (D) (i.e., 250×CUP1RU and 380×CUP1RU strains) were used.

Rtt109 is the sole enzyme responsible for acetylation at Lys-56 of histone H3.^21^ Accordingly, the absence of H3K56ac in *rtt109*Δ strains likely accelerates BITREx while inducing marked heterogeneity, indicative of potential instability (Figures 2J and S6H). Considering that the stability of expanded arrays is crucial for practical applications, we decided to further investigate the role of H3K56ac. For this purpose, we examined the effect of nicotinamide (NAM), which inhibits the NAD^+^-dependent histone deacetylase family consisting of Sir2, Hst1, Hst2, Hst3, and Hst4 in the budding yeast.^22^ Previous studies have reported that NAM induces *CUP1* copy-number reduction in an Rtt109/H3K56ac-dependent manner.^8^^,^^23^ We first confirmed that H3K56ac accumulates in the 14×CUP1RU strain after 24 h of NAM exposure (Figure 3A). Notably, the presence of NAM not only suppressed BITREx of the *CUP1* array (Figure 3B), but also led to its gradual contraction, regardless of whether nCas9 was targeted to the *CUP1*-flanking site or the control site (Figure 3C). The effect of NAM on highly extended *CUP1* arrays was remarkable: the 250×CUP1RU and 380×CUP1RU strains showed a drastic decrease in the copy number during 3 days of NAM exposure (Figure 3D).

These results suggested that the effect of NAM on CNA depends on the initial length of the *CUP1* array. To test this hypothesis, we prepared a series of cell populations with different average *CUP1* copy numbers by temporal sampling from a long-term BITREx culture. Each sample was divided into two subpopulations, which were then cultured in parallel in the presence and absence of NAM for 3 or 4 days (Figure 3E). In all samples, NAM exposure induced a steep decrease in *CUP1* copy number, converging to <30 copies (Figure 3F). There was no difference in CNA/G levels with or without nCas9 induction (Figure 3G), likely because NAM efficiently suppresses BITREx (Figure 3B). The CNA/G appeared to inversely correlate with the initial *CUP1* copy number (Figure 3H), as expected from the theoretical model, which assumes array contraction via homologous recombination between repeat units following second-order kinetics (see STAR Methods for details).

Note that the effects of NAM on BITREx and the *CUP1* array were completely abolished in the absence of Rtt109, the sole H3K56 acetylase (Figures 3A, 3B, and 3D). These results indicate that, although NAM leads to the accumulation of acetylation at multiple Lys residues, its effects are mediated through the accumulated H3K56ac.

### General applicability of BITREx

**Figure 4:**
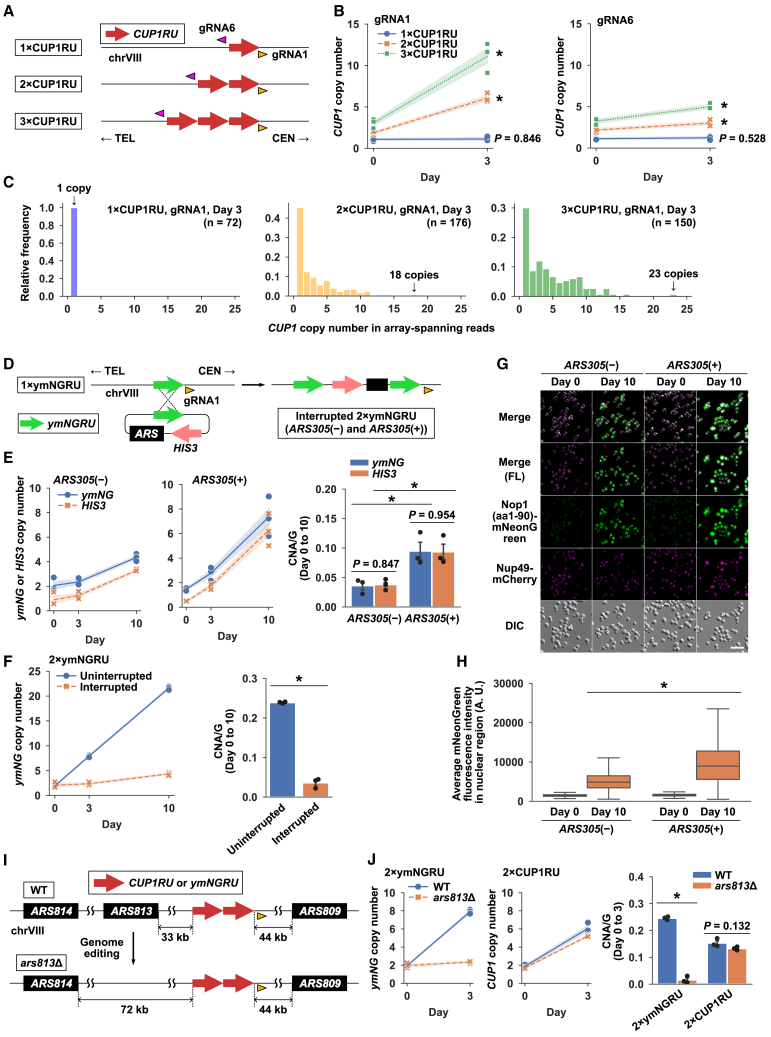
Core requirements and modulating factors for BITREx (A) Schematic of the strains with one to three copies of *CUP1RU*. Orange and magenta arrowheads indicate the target sites of gRNA1 and gRNA6, respectively. (B) *CUP1* CNA in the three strains depicted in (A). Shading, SD (*n* = 3 biological replicates). ∗*p* < 0.05 (Student’s t test). (C) Distribution of *CUP1* copy number in nanopore reads spanning the entire array. Nanopore sequencing was performed on day 3 of BITREx. (D) Plasmid integration strategy to generate an interrupted *2×ymNGRU* array. The intervening sequence was composed of the plasmid backbone containing *HIS3* with or without *ARS305*. (E) CNA of *ymNGRU* and *HIS3* in the absence and presence of the embedded *ARS305*. Shading and error bar, SD (*n* = 3 biological replicates). ∗*p* < 0.05 (Student’s t test). (F) CNA of *ymNGRU* in the uninterrupted and interrupted two-unit arrays. Shading and error bar, SD (*n* = 3 biological replicates). ∗*p* < 0.05 (Student’s t test). (G) Microscopic images of strains bearing the interrupted *2×ymNGRU* arrays without and with the embedded *ARS305*. These strains have *NUP49-mCherry* to visualize the nuclei (magenta). FL, fluorescence; DIC, differential interference contrast. Scale bar, 20 μm. (H) Quantification of mNeonGreen fluorescence in (G). Boxplots indicate the distribution of the average mNeonGreen fluorescence intensity in the nuclear region. The bottom and top of the box show the first and third quartiles, respectively. The bar in each box represents the median value, and the error bars represent the range of values. ∗*p* < 0.001 (one-way ANOVA test). (I) ARS distribution around the *CUP1* locus in the wild-type and *ars813*Δ strain. Arrowhead, gRNA1 target site. (J) Effect of *ARS813* on BITREx of the uninterrupted *2×ymNGRU* and *2×CUP1RU* arrays. The *2×ymNGRU* array lacks internal ARS, while the *2×CUP1RU* array contains *ARS810/811* within the repeat unit. Shading and error bar, SD (*n* = 3 biological replicates). ∗*p* < 0.05 (Student’s t test).

Theoretically, BITREx can extend a tandem array consisting of two or more repeat units but not a single-copy unit. We constructed strains harboring *CUP1* arrays consisting of one, two, and three repeat units (i.e., 1×CUP1RU, 2×CUP1RU, and 3×CUP1RU) to determine the minimum number of repeat units required for BITREx (Figure 4A). When targeted to an upstream or downstream flanking site of the *CUP1* array for 3 days, nCas9 increased the *CUP1* copy number in the 2×CUP1RU and 3×CUP1RU strains, but not in the 1×CUP1RU strain (Figure 4B). Nanopore sequencing revealed the expanded *CUP1* arrays in the 2×CUP1RU and 3×CUP1RU strains but not in the 1×CUP1RU strain (Figure 4C). All these results are consistent with the BIR-based mechanism of BITREx.

In addition, we found that BITREx is applicable to the non-*CUP1* natural tandem gene array *ENA1/2/5* encoding P-type ATPase sodium pumps^24^^,^^25^^,^^26^ (Figures S7A–S7E); a synthetic two-unit array composed of a fusion gene between *NOP1*^27^ and *mNeonGreen* (*2×ymNGRU*) at the *CUP1* locus (Figures S7F–S7I); and two-unit arrays, with or without intervening sequences, integrated at loci that do not naturally form tandem repeats (*HO* and *X-2*)^28^^,^^29^ (Figures S8A–S8F).

Taken together, BITREx can expand two-unit arrays, whether uninterrupted or interrupted, at different genomic loci. However, the efficiency of expansion varies depending on the locus and the composition of the array.

### Effects of ARS on BITREx

The successful expansion of the interrupted two-unit arrays prompted us to explore the possibility of BITREx-mediated amplification of a target sequence embedded between two repeat units, as this configuration can be easily generated using the conventional plasmid integration technique. For example, we integrated a ∼5-kb plasmid carrying *ymNGRU* and *HIS3* into the single-copy *ymNGRU* at the *CUP1* locus (Figure 4D) and successfully applied BITREx to the resulting interrupted two-unit array, leading to a simultaneous increase in the copy numbers of *ymNGRU* and *HIS3* (Figure 4E). However, the interrupted *2×ymNGRU* array showed a significantly lower CNA/G than the uninterrupted *2×ymNGRU* array (Figure 4F). Given the importance of replication fork directionality (Figures S8A–S8C), we were concerned that the ∼3.4-kb plasmid-derived intervening sequence might have decreased the likelihood of achieving the desired fork direction at the nick. To mitigate such adverse effects, we incorporated an autonomously replicating sequence (ARS) into the repeat unit. As expected, incorporating *ARS305* effectively increased CNA/G and enhanced the fluorescence (Figures 4E, 4G, and 4H).

These results led us to hypothesize that BITREx of a tandem gene array without an internal ARS would be sensitive to the distance between the nick on one side and the nearest ARS on the opposite side of the array. In contrast, BITREx of a tandem gene array with an ARS within the repeat unit would not be affected by this distance. To test this hypothesis by elongating the distance between the nick and the ARS, we generated strains deleted for *ARS813*, the nearest ARS responsible for BITREx using gRNA1 at the *CUP1* locus (Figure 4I). Indeed, BITREx of the *2×ymNGRU* array, which lacks an internal ARS, depended on *ARS813*, while BITREx of the *2×CUP1RU* array, which includes *ARS810/811*, did not (Figure 4J). The incorporation of an ARS had no negative effect on BITREx and even enhanced it. Therefore, it is desirable to incorporate an ARS in the repeat unit, especially when no suitable external ARS is available near the target locus or when the repeat unit is long.

Based on these results, we attempted to amplify a multigene array using BITREx. We used plasmid integration to construct strains where the two-unit *CUP1* array at the *CUP1* locus was interrupted by an ∼8.5-kb fragment containing yeast codon-optimized coding sequences for four fluorescent proteins (mTagBFP, miRFP682, mCherry, and mNeonGreen) and *HIS3*, with and without *ARS305* (Figure S8G). BITREx successfully extended the array, enhancing gene dosage effects, particularly in the strain with *ARS305* in the repeat unit (Figures S8H–S8J), highlighting the potential of this approach for overexpressing useful gene products.

### Splinted BITREx for *de novo* generation of tandem gene arrays

The minimum requirement for BITREx is two identical sequences to form either an uninterrupted or interrupted two-unit array. However, because BIR frequently switches templates,^30^ we hypothesized that BITREx could generate a tandem array starting from a single-copy sequence if an engineered DNA serves as a “splint” to mediate the necessary template switching.

**Figure 5:**
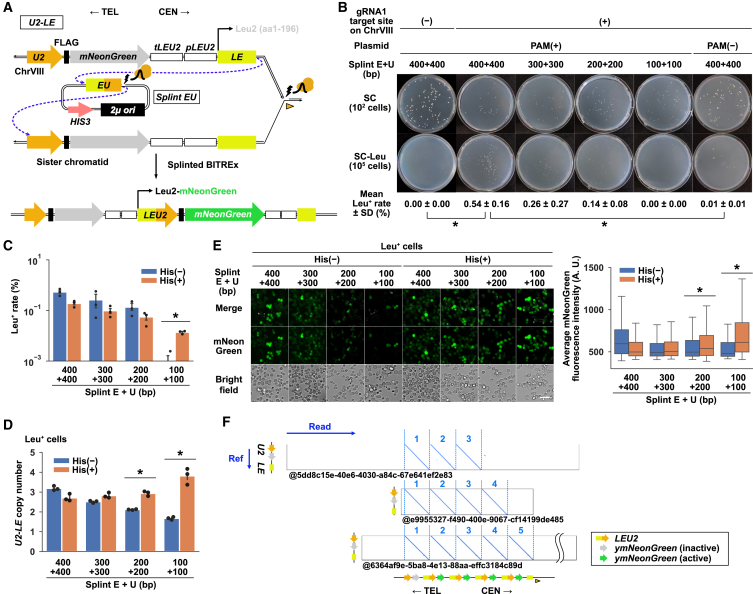
*De novo* generation of tandem gene array by splinted BITREx (A) Proof-of-concept experiment for splinted BITREx. A single round of splinted BITREx reconstitutes the *LEU2*-coding sequence, resulting in the expression of a fusion protein composed of Leu2 and mNeonGreen connected by the FLAG tag. *tLEU2*, *LEU2* terminator; *pLEU2*, *LEU2* promoter; orange arrowhead, gRNA1 target site. (B) Efficiency of splinted BITREx assessed by the appearance of Leu^+^ clones. Splints of four different lengths were tested over 3 days of BITREx. Representative images show colonies formed on SC and SC-Leu agar plates, inoculated with approximately 10^2^ and 10^5^ cells, respectively. The mean Leu^+^ rate with SD is shown at the bottom of the panel (*n* = 3 biological replicates). ∗*p* < 0.05 (Student’s t test). (C) Effects of histidine supplementation from day 2 on the appearance of Leu^+^ colonies. Error bar, SD (*n* = 3 biological replicates). ∗*p* < 0.05 (Student’s t test). (D) Effects of histidine supplementation from day 2 on the copy number of *U2-LE* unit. Error bar, SD (*n* = 3 biological replicates). ∗*p* < 0.05 (Student’s t test). (E) Effects of histidine supplementation from day 2 on the mNeonGreen fluorescence. Left, microscopic images of strains subjected to splinted BITREx. Scale bar, 20 μm. Right, boxplots showing the distribution of the average mNeonGreen fluorescence intensity in cells. ∗*p* < 0.05 (Student’s t test). (F) Dot plot comparing nanopore reads to the reference sequence of *U2-LE* unit.

To test this idea, we constructed a strain that harbors a repeat unit delimited by *U2* and *LE* (two non-overlapping consecutive fragments of the *LEU2* coding sequence) on chromosome VIII and a plasmid carrying the splint fragment *EU* (Figure 5A). Upon the induction of a seDSB by targeting nCas9 to a downstream-flanking site of the genomic *LE*, the ssDNA generated by end-resection of the break site may use its *E* to invade the episomal *EU* for further extension by displacement synthesis. The extended ssDNA may then use the newly acquired *U* to switch its template from the splint plasmid to the genomic *U2*. If these consecutive strand invasion-extensions occur, the *U2-LE* unit duplicates to reconstitute the *LEU2* gene, allowing the growth in the absence of leucine. Indeed, we observed the emergence of Leu^+^ cells with different efficiencies depending on the length of the splint fragment (Figure 5B). Notably, co-nicking of the chromosome and the splint plasmid was essential for the *LEU2* reconstitution (Figure 5B).

Once the *LEU2* gene is reconstituted, the *LE* ssDNA can initiate BIR either by directly invading the reconstituted *LEU2* or indirectly invading the genomic *U2* or *LEU2* via the splint plasmid (Figure 5A). Since the direct invasion should be much more efficient than the indirect invasion, the splint plasmid may inhibit subsequent cycles of BITREx, even though it is essential for the initial reconstitution of *LEU2*. Based on these considerations, we examined the effect of histidine supplementation starting from day 2 to promote the spontaneous loss of the *HIS3*-marked splint plasmid. This supplementation protocol improved the emergence of Leu^+^ clones when combined with the shortest splint fragments (Figure 5C). It also increased the copy number of *U2-LE* units and the mNeonGreen fluorescence when combined with the shortest or second shortest splint fragment (Figures 5D and 5E). Nanopore sequencing identified reads spanning a tandem array composed of up to five *U2-LE* units or four *LEU2-mNeonGreen*-fusion genes (Figure 5F).

These results demonstrated that “splinted BITREx” enables *de novo* formation of a tandem gene array from a single-copy sequence.

### BITREx in mammalian cells

**Figure 6:**
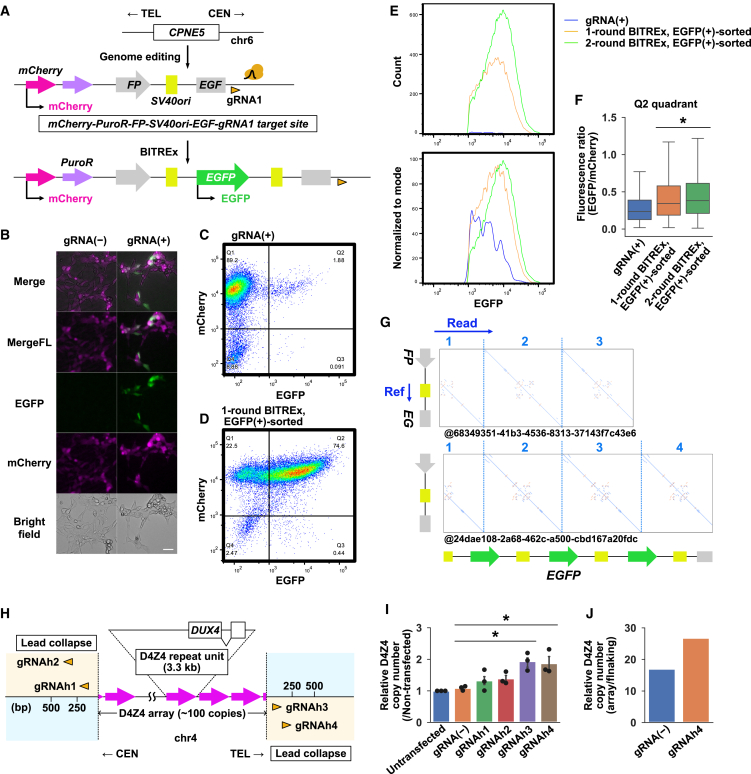
BITREx in mammalian cells (A) Proof-of-concept experiment for BITREx in HEK293T cells using an *EGFP* reconstitution reporter integrated into the *CPNE5* locus. (B) Microscopic images of HEK293T cells with the integrated reporter on day 4 after transfection of the nCas9-gRNA1 expression plasmid. Scale bar, 50 μm. (C) Flow cytometric analysis of mCherry and EGFP expression on day 3 after transfection of the nCas9-gRNA1 expression plasmid. The cells gated based on light scatter (Figure S9C) displayed mCherry and EGFP expression across four quadrants (Q1, Q2, Q3, Q4), with the percentage distribution indicated in each quadrant. (D) Flow cytometric analysis of mCherry and EGFP expression in the mCherry^+^/EGFP^+^ cells sorted from quadrant Q2 in (C). (E) Distribution of EGFP fluorescence. Blue, the total population of cells transfected with the gRNA1-nCas9 co-expression plasmid; orange, flow-sorted EGFP^+^ cells; green, flow-sorted EGFP^+^ cells on day 3 after re-transfection with the co-expression plasmid. The x axis represents EGFP fluorescence intensity, while the y axis represents either the number of cells (top) or the value normalized to mode (bottom). (F) Boxplots showing the fluorescence ratio of EGFP to mCherry in the cells in (E). ∗*p* < 0.05 (Student’s t test). (G) Dot plots comparing nanopore reads to the reference sequence of the *FP-EG* unit. Nanopore whole-genome sequencing identified 38 reads that included the *FP-EG* unit, 26 of which supported the expected expansion. The upper and lower reads indicate at least triplication and quadruplication, respectively. (H) Schematic of the D4Z4 array and its flanking regions on human chromosome 4. Arrowheads indicate the target sites of four gRNAs, which guide nCas9 to induce lead collapse of the replication fork moving outward from the D4Z4 array. (I) D4Z4 copy number quantified by qPCR on day 3 of BITREx with the indicated gRNA. Data from transfected cells were normalized to untransfected control cells. Error bar, SD (*n* = 3 biological replicates). ∗*p* < 0.05 (Student’s t test). (J) Ratio of normalized read counts between the D4Z4 macrosatellite and the centromeric flanking region.

We next investigated the feasibility of BITREx in mammalian cells. Since BIR has been demonstrated in mammalian cells using the reconstitution of a fluorescent protein gene,^31^ we generated a reporter construct *mCherry-PuroR-FP-SV40ori-EGF-gRNA1 target site* (Figure 6A). In this construct, the *mCherry-PuroR* portion serves as a transfection reporter/marker, while the *FP-SV40ori-EGF* portion serves as an interrupted two-unit array, in which two *F* fragments are interrupted by the *P-SV40ori-EG* fragment. Therefore, nCas9 with gRNA1 should induce BIR via the *F* fragment to reconstitute the *EGFP* gene, resulting in green fluorescence.

We initially aimed to integrate the construct to the safe harbor locus *AAVS1* of human HEK293T cells, which expresses the large T antigen that activates *SV40ori*, the replication origin of the SV40 virus, using the VIKING method for efficient NHEJ-based knockin (Figure S9A).^32^ However, genotyping and nanopore sequencing of the puromycin-selected cells revealed that the construct had been integrated not into *AAVS1* but into *CPNE5*, a previously reported off-target site for the *AAVS1* gRNA we used (Figure S9B).^33^ Despite this, these cells provided a valuable opportunity to investigate the feasibility of BITREx in mammalian cells. To this end, we transfected them with a plasmid that co-expresses nCas9 and gRNA1 to induce BITREx (Figure S9A). As a fraction of the transfected cells started to show EGFP fluorescence (Figure 6B), we flow-sorted the mCherry^+^/EGFP^+^ cells (Figures 6C, 6D, and S9C). A subsequent round of transfection with the co-expression plasmid conferred enhanced EGFP fluorescence to the cells (Figure 6E). As expected from the design of the reporter construct, in which BITREx increases the copy number of the *EGFP* gene but not the *mCherry* gene (Figure 6A), the EGFP/mCherry fluorescence ratio increased (Figures 6F and S9D). Nanopore sequencing of these cells identified reads containing at least three or four copies of the *FP-SV40ori-EG* unit (Figure 6G). These results demonstrated the feasibility of BITREx in mammalian cells.

Note that we cannot rule out the possibility that the results described above were influenced by residual plasmids encoding the reporter construct in the puromycin-selected cells (Figure S9E). Therefore, we sought to apply BITREx to gene-sized tandem repeats naturally occurring in the human genome. For this purpose, we focused on the D4Z4 array, which consists of a 3.3-kb repeat unit on chromosome 4q35. This array is significant because its heterozygous contraction causes facioscapulohumeral muscular dystrophy (FSHD), the third most common type of inherited muscular dystrophy.^34^ Accordingly, its expansion may have potential implications in FSHD therapeutics. We designed four gRNAs to target nCas9 to the centromeric and telomeric sides of the D4Z4 array (Figure 6H). The results of qPCR consistently demonstrated that targeting nCas9 to the telomeric side increased the D4Z4 copy number (Figure 6I). We subjected the cells with no gRNA and the most effective gRNA (gRNAh4) to nanopore sequencing. The normalized read count indicated that the D4Z4 copy number increased from ∼17 to ∼27 copies (Figure 6J), consistent with the qPCR results (Figure 6I). Although the complexity of the D4Z4 locus containing many repetitive sequences and the presence of an almost identical locus on chromosome 10q26 precluded the complete characterization of the induced CNAs, these results demonstrated the applicability of BITREx to endogenous tandem gene arrays in mammalian genomes.

## Discussion

We have developed BITREx, a method for expanding tandem gene arrays through continuous ectopic BIR induced by strategically targeting nCas9. In BITREx, nCas9 is placed adjacent to a tandem gene array, disrupting the replication fork after it has replicated the array. We previously developed paired nicking-induced amplification (PNAmp), a method for inducing large segmental duplications by paired nicking-induced SSA.^35^ Similarly, others have developed methods using a pair of prime editors, although the underlying mechanisms remain somewhat unproven.^36^^,^^37^ While both PNAmp and BITREx induce structural variations by manipulating replication fork progression, they are mechanistically distinct: PNAmp uses two gRNAs and depends on SSA,^35^ whereas BITREx uses one gRNA and relies on BIR. The basic requirements for a PNAmp target are an internal ARS and terminal direct repeats, or an interrupted two-unit array with an ARS.^35^ This is because both nicks at the flanking sites of the target segment must be crossed by outward replication forks. In contrast, BITREx can be applied to an interrupted two-unit array even without an ARS, as it can be designed to work as long as at least one of the flanking sites is crossed by an outward replication fork. Therefore, BITREx is suitable for a broader range of targets compared with PNAmp.

For BITREx to be effective, the replication fork crossing the nick must move from inside to outside the tandem gene array. If the repeat unit lacks an ARS, BITREx depends on replication initiated from an external ARS flanking the tandem gene array on the opposite side of the induced nick. As the array expands, the distance between the ARS and the nick increases, thus decreasing the likelihood that the outward replication fork will reach the nick earlier than the inward fork initiated from the nearest ARS on the same side as the nick. Consequently, the success rate of BITREx per cell cycle declines, reaching a plateau to limit the maximum expansion range. In contrast, if the repeat unit contains an ARS, cells can more reliably maintain the desired replication fork direction. The internal ARS makes BITREx more autonomous and less dependent on an external ARS. Therefore, including an ARS in the repeat unit of a synthetic array is advantageous. Although autonomous BITREx theoretically permits unlimited expansion, the increased instability of highly extended arrays appears to counteract this potential, leading to a plateau where expansion by BITREx is balanced by contraction due to intrinsic instability. Moreover, prolonged nicking can occasionally induce mutations at the target site, leading to progenies that are free from the nCas9-induced seDSB: these progenies should outcompete those with the seDSB in proliferation speed, eventually dominating the cell population. These factors likely impose practical limits on the extent of expansion.

The efficiency of BITREx is influenced by multiple factors, as demonstrated by the CNA/G values for six two-unit arrays integrated at three different loci (Table S1). Arrays embedded at the same locus exhibited varying efficiencies, emphasizing the importance of repeat unit characteristics. Conversely, identical arrays behaved differently depending on their integration loci, highlighting the influence of the local genomic environment. Collectively, these results suggest that the overall efficiency of BITREx is governed by a complex interplay of factors, making precise predictions inherently challenging.

Interestingly, BITREx requires nCas9 to nick the template DNA for leading strand synthesis, but not for lagging strand synthesis. This strand specificity likely reflects the asymmetry observed in the repair of replication-coupled DNA breaks, as revealed in recent studies on mammalian cells: nicks on the leading and lagging strand templates lead to the formation of seDSBs and double-ended DSBs (deDSBs), respectively.^38^^,^^39^ The replisome notably bypasses nicks on the lagging strand templates to generate deDSBs directly, or without contribution from the converging replication fork, thereby likely preventing BIR. Intriguingly, one yeast study showed that a nick on a leading strand template induces a seDSB, while another nick on a different leading strand template induces a deDSB, independently of the converging replication fork.^40^ Although the determinants of these differential fates remain elusive, these variations may partly explain why 10 out of the 16 gRNAs designed for lead collapse were ineffective.

While BITREx increased the average copy number of repeat units in the population, nanopore sequencing revealed the presence of contracted arrays. We hypothesized that extensive 5′-to-3′ end-resection and/or the “chewing back” of the invading strand by the 3′-to-5′ exonuclease activity of Pol δ^41^ contribute to the contraction (Figures S4A and S4B). Therefore, appropriate suppression of these end resection activities may prevent contraction events and improve BITREx. These situations are more likely to occur when the repeat unit is short, meaning BITREx may not be effective in expanding micro- and mini-satellite DNAs, particularly when the repeat number is low. However, BITREx could still be valuable for extending satellite DNA-like repeats, enabling the synthesis of centromere-like DNA—an ongoing challenge in synthetic genomics.

An obvious application of BITREx would be the overexpression of genes of interest. We have successfully applied BITREx to an interrupted two-unit array containing four fluorescent protein genes as the intervening sequence, resulting in their overexpression to exert a dosage effect. These results suggest that when applying to a two-unit array with a biosynthetic gene cluster as the intervening sequence, BITREx can enhance the yield of the biosynthetic pathway’s product. In contrast to the serial configuration within a gene cluster, individual genes encoding pathway components can be distributed across multiple loci, where BITREx could act in parallel to increase their copy numbers. BITREx occurs stochastically in each cell cycle. Once it occurs, the subsequent cell division becomes asymmetric regarding unit copy number: one daughter cell inherits the donor chromatid with the original array, while the other inherits the acceptor chromatid with the expanded array. In addition, the efficiency of BITREx varies from one locus to another. Therefore, parallel BITREx would create a cell population with diverse stoichiometry among pathway components, which could help identify an optimal pathway design for maximizing the yield of the pathway’s product.

It should be noted that BIR is less accurate than normal S-phase replication.^42^ During BIR, the Pif1 helicase immediately dissociates the newly synthesized leading strand DNA from its template, leaving it single-stranded until lagging strand synthesis occurs (Figure S1B). As a result, the mismatch repair system functions ineffectively in BIR. Furthermore, the exposed nucleobases in ssDNA are much more susceptible to damage compared with those in double-stranded DNA. Indeed, inducing BIR in yeast in the presence of an alkylating agent has led to the formation of mutation clusters similar to those found in cancer genomes.^43^ Therefore, we hypothesize that BITREx in the presence of mutagens, or mutagenic BITREx, will not only expand a tandem gene array but also diversify its repeat unit sequence, potentially generating an array of paralogs similar to OR gene loci. Moreover, gap repair cloning can be employed to randomly isolate individual paralogs and place them under a promoter, producing a unique population of cells in which each clone expresses a distinct paralog, mirroring the diversity seen in olfactory neurons or lymphocytes. These diverse cell populations hold promise for unique applications in both basic research and applied studies.

It is also noteworthy that BITREx may represent the first method for the targeted expansion of macrosatellite repeats with medical or therapeutic significance, such as D4Z4, DXZ4, and the exon array in the *LPA* gene.^34^^,^^44^^,^^45^ This approach could advance mechanistic studies of macrosatellite-associated diseases and potentially contribute to the development of regenerative therapies.

We anticipate that BITREx will enable these and other unique applications in genome engineering.

### Limitations of the study

BITREx is a replication-coupled process and thus not applicable to non-dividing cells. By its nature, it cannot effectively expand tandem gene arrays located between actively firing nearby replication origins. Currently, predicting the performance of BITREx is challenging due to its reliance on various factors, including gRNA efficacy in inducing seDSB, the local environment of the target locus, and the composition of the repeat unit sequence, as some sequences can hinder BIR.^46^ In addition, there is no method for stably maintaining tandem gene arrays that have been highly expanded by BITREx. Further investigations are required to optimize BITREx in mammalian cells, including analyses of the temporal dynamics of array expansion and stability, which are expected to differ from those observed in yeast.

## Resource availability

### Lead contact

Further information and requests for resources and reagents should be addressed to and will be fulfilled by the lead contact, Takashi Ito (ito.takashi.352@m.kyushu-u.ac.jp).

### Materials availability

Requests for the generated plasmids and strains in this study should be directed to the lead contact, Takashi Ito (ito.takashi.352@m.kyushu-u.ac.jp).

### Data and code availability

• All raw sequencing data used in this study were deposited in the DDBJ BioProject database: PRJDB18647, PRJDB18687, PRJDB18705.
• All original codes used in this study are available from Zenodo at https://doi.org/10.5281/zenodo.11515696.

## Acknowledgments

We thank Tetsuya Hayashi and Yasuhiro Gotoh for the PFGE equipment. We appreciate the technical assistance from the Research Support Center of the Research Center for Human Disease Modeling at Kyushu University Graduate School of Medical Sciences, which is partially supported by the Mitsuaki Shiraishi Fund for Basic Medical Research. This work was funded by JST CREST grant no. JPMJCR19S1 and 10.13039/501100001691JSPS KAKENHI grant no. 24K02015.

## Author contributions

Conceptualization, H.T., S.O., and T.I.; methodology, H.T., S.O., and G.D.; investigation, H.T. and S.O.; writing – original draft, H.T.; writing – review & editing, H.T., S.O., and T.I.; funding acquisition, T.I.; resources, H.T., S.O., G.D., Y.S., and E.K.; supervision, T.I.

## Declaration of interests

The authors declare no competing interests.

## Declaration of generative AI and AI-assisted technologies

During the preparation of this work, the authors used ChatGPT to improve the readability of certain sentences. After using this tool/service, the authors reviewed and edited the content as needed and take full responsibility for the content of the publication.

## STAR★Methods

### Key resources table

**Table:** 

REAGENT or RESOURCE	SOURCE	IDENTIFIER
**Antibodies**		

Anti-Histone H3 Antibody, CT, pan, clone A3S, rabbit monoclonal antibody	Sigma-Aldrich	Cat# 05-928 RRID:AB_492621
Histone H3K56ac rabbit polyclonal antibody	Active Motif	Cat# 39281 RRID:AB_2661786
Anti-rabbit IgG, HRP-linked Antibody	Cell Signaling Technology	Cat# 7074 RRID:AB_2099233

**Bacterial and virus strains**		

DH5α high Champion™ cell	SMOBIO	Cat# CC5202

**Chemicals, peptides, and recombinant proteins**		

17β-Estradiol	Nakarai tesque	Cat# 14541-74
Nicotinamide	Nakarai tesque	Cat# 24317-72

**Critical commercial assays**		

KOD One® PCR Master Mix (Dye-free 2×PCR Master Mix)	TOYOBO	Cat# KMM-101
KOD SYBR® qPCR Mix	TOYOBO	Cat# QKD-201
Chelex 100 Chelating Resin, biotechnology grade, 100–200 mesh, sodium form	Bio-Rad	Cat# 1432832
Quick-DNA Fungal/Bacterial Miniprep Kit	ZYMO RESEARCH	Cat# D6005
NucleoSpin Tissue	TaKaRa	Cat# 740952.50
QIAfilter Plasmid Midi Kit	QIAGEN	Cat# 12243
Monarch HMW DNA Extraction Kit for Cell & Blood	NEB	Cat# T3050L
Monarch HMW DNA Extraction Kit for Tissue	NEB	Cat# T3060L
NEB Golden Gate Assembly Kit (BsaI-HF v2)	NEB	Cat# E1601L
NEBuilder HiFi DNA Assembly Master Mix	NEB	Cat# E2621L
ThruPLEX DNA-Seq kit	TaKaRa	Cat# R400674
DNA Single Index kit – 12S Set A	TaKaRa	Cat# R400695
DNA Single Index kit – 12S Set B	TaKaRa	Cat# R400697
MiSeq Reagent Kit v3 (150-cycle)	Illumina	Cat# MS-102-3001
Certified Megabase Agarose	Bio-Rad	Cat# 1613108
CHEF Genomic DNA Plug Kits	Bio-Rad	Cat# 1703491
12% Mini-PROTEAN® TGX™ Precast Protein Gels, 12-wells	Bio-Rad	Cat# 4561045
SYBR Green I Nucleic Acid Gel Stain	Thermo-Fisher	Cat# S7563
Trans-Blot Turbo Mini 0.2μm PVDF Transfer Packs	Bio-Rad	Cat# 1704156
iBind™ Solution Kit	Thermo Fisher	Cat# SLF1020
iBind™ Cards	Thermo Fisher	Cat# SLF1010
Clarity Max Western ECL Substrate	Bio-Rad	Cat# 1705062
AlkPhos Direct Labeling Module for 25 labellings	Cytiva	Cat# RPN3680
CDP-Star Detection Reagent for 2,500 cm^2^ membrane	Cytiva	Cat# RPN3682
AlkPhos Direct Hybridization Buffer for 5,000 cm^2^ membrane	Cytiva	Cat# RPN3688
Lipofectamine 3000® Reagent	Thermo Fisher	Cat# L3000008
Quick-DNA Microprep Kit	ZYMO RESEARCH	Cat# D3020
Ligation Sequencing Kit	Oxford Nanopore Technologies	SQK-LSK109
Ligation Sequencing Kit	Oxford Nanopore Technologies	SQK-LSK114
Native Barcoding Expansion 1-12	Oxford Nanopore Technologies	EXP-NBD104
Native Barcoding Expansion 13-24	Oxford Nanopore Technologies	EXP-NBD114
Native Barcoding Kit 96 V14	Oxford Nanopore Technologies	SQK-NBD114.96
MinION Flow Cell (R9.4.1)	Oxford Nanopore Technologies	FLO-MIN106D
PromethION Flow Cell (R10.4.1)	Oxford Nanopore Technologies	FLO-PRO114M
Flongle Flow Cell (R10.4.1)	Oxford Nanopore Technologies	FLO-FLG114

**Deposited data**		

*S. cerevisiae* S288C reference genome: sacCer3	Saccharomyces Genome Database	https://www.ncbi.nlm.nih.gov/datasets/genome/GCF_000146045.2/
Genome assembly T2T-CHM13v2.0	T2T Consortium	https://www.ncbi.nlm.nih.gov/datasets/genome/GCF_009914755.1/
Raw sequence data	This paper	DDBJ BioProject database: PRJDB18647, PRJDB18687, PRJDB18705

**Experimental models: Cell lines**		

HEK293T	RIKEN BRC	RBRC-RCB2202

**Experimental models: Organisms/strains**		

*S. cerevisiae*: Strain background: BY4741	N/A	N/A
All other synthetic yeast strains used in this paper, listed in Table S2	This paper	N/A

**Oligonucleotides**		

All oligonucleotides used in this paper, listed in Table S3	This paper	N/A

**Recombinant DNA**		

pyChrIV545kb_FP-SV40ori-EGF-yChrIV592kb_RFP_puroR	Sugiyama et al.^35^	N/A
VKG1-gRNA-pX330	Sawatsubashi et al.^32^	Addgene plasmid #108671
AAVS1 T2 CRISPR in pX330	Natsume et al.^47^	Addgene plasmid #72833
AIO-Puro	Chiang et al.^48^	Addgene plasmid #74630
All other plasmids used in this paper, listed in Table S4	This paper	N/A

**Software and algorithms**		

MinKNOW	Oxford Nanopore Technologies	https://community.nanoporetech.com/downloads?from=support
Guppy v6.5.7	Oxford Nanopore Technologies	https://community.nanoporetech.com/downloads?from=support
Dorado v0.7.3	Oxford Nanopore Technologies	https://community.nanoporetech.com/downloads?from=support
NanoPlot	De Coster et al.^49^	https://github.com/wdecoster/NanoPlot
YASS	Noé and Kucherov^50^	https://bioinfo.univ-lille.fr/yass/index.php
Minimap2 v2.17-r941	Li^51^	https://github.com/lh3/minimap2
samtools v1.10	Danecek et al.^52^	https://github.com/samtools/samtools
bedtools v2.27.1	Quinlan and Hall^53^	https://github.com/arq5x/bedtools2
Read_split_by_target.py	Satoshi Okada	https://doi.org/10.5281/zenodo.11515698
Target_seq_extraction_single.py	Satoshi Okada	https://doi.org/10.5281/zenodo.11515698
Bedgraph_norm_ratio.py	Satoshi Okada	https://doi.org/10.5281/zenodo.11515696
minialign	Hajime Suzuki	https://github.com/ocxtal/minialign
BLAST	Altschul et al.^54^	https://blast.ncbi.nlm.nih.gov/blast/Blast.cgi
Bowtie2 v2.3.5	Langmead and Salzberg^55^	https://github.com/BenLangmead/bowtie2

### Experimental model and subject details

The budding yeast *Saccharomyces cerevisiae* was used as the primary experimental model in the study. The haploid yeast strain BY4741^56^ was used as the parental strain. As a model of mammalian cells, the human female embryonic kidney-derived cell line HEK293T was purchased from RIKEN BRC (catalog number RBRC-RCB2202) and cultured in Dulbecco’s modified Eagle medium (DMEM) (Gibco, catalog number 11885084) at 37°C.

### Method details

#### Yeast strains

All yeast strains used in this study are derived from BY4741 (*MAT***a** *his3*Δ1 *leu2*Δ0 *met15*Δ0 *ura3*Δ0)^56^ (Table S2). This study used standard culture media and genetic methods.^57^ We deleted a gene of interest by transforming yeast cells with a DNA fragment composed of a *KanMX* cassette sandwiched by the 5′- and 3′-flanking sequences of the open reading frame of the gene, which was amplified from the corresponding deletant strain in Yeast Deletion Clones *MAT***a** Complete Set (Invitrogen) using PCR primers listed in Table S3.

#### Yeast plasmids

All plasmids used in this study are listed in Table S4. All primers for plasmid construction were purchased from Sigma-Aldrich and Eurofins Genomics. Plasmids were constructed by seamless cloning using HiFi DNA Assembly (NEB) or Golden Gate Assembly (NEB).

The integrative plasmid YIplac128-pGAL1-nCas9 (Cas9^D10A^ or Cas9^H840A/N854A^)-tADH1 (*LEU2*) harbors a gene encoding nCas9 derived from *Streptococcus pyogenes* fused with the SV40 nuclear localization signal as described previously^58^ under the control of the *GAL1* promoter. It was used for yeast transformation after AgeI digestion to be integrated into the *GAL1* promoter on the genome.

The integrative plasmid pFA6a-pCUP2-yGEV-tADH1-HphMX (HygR) harbors a gene encoding β-estradiol-responsive artificial transcription activator GEV^14^ under the control of the *CUP2* promoter. It was used for yeast transformation after MfeI digestion to be integrated into the *CUP2* promoter on the genome.

Centromeric plasmids for gRNA expression harbor a gRNA gene under the control of the *GAL1* promoter. The gRNA scaffold sequence contains a base-flip and a stem-loop extension for stable gRNA expression.^59^ To cut off an unnecessary sequence from the 5′-terminal portion of the gRNA-containing transcript, each gRNA sequence is preceded by a hammerhead ribozyme (Table S5). To define the 3′-terminus, each gRNA sequence is followed by the HDV ribozyme on the *GAL1* promoter plasmid (Table S5). For designing gRNAs, CRISPRdirect^60^ was used to select target sites in the yeast genome listed in Table S5.

#### Yeast genome editing

For constructing the *gRNA1inv*, *pif1-m2*, *rtt109-K290Q*, *cup1ru*Δ:*ymNG* array, *gRNA1ts-cup1ru*Δ::*NatMX*, *ho*Δ:2×*CUP1RU*, *ho*Δ:2×*ymNGRU, X-2*Δ:2×*CUP1RU*, *X-2*Δ:2×*ymNGRU* strains, we performed SpCas9 or enAsCas12a-based gene editing as described previously.^61^ All gene-editing plasmids used in this study are listed in Table S4.

#### Yeast cell culture

Yeast cells were grown at 30°C overnight in 2 mL of SC−Ura, SC−His−Ura, SC−Leu−Ura, or SC−His−Leu−Ura medium supplemented with 2% glucose with or without G418 disulfate and/or hygromycin B (Nakalai tesque). On the following day, the OD_620_ of each sample was recorded, and 10–50 μL of the culture diluted up to 1 × 10^6^ times was inoculated into 2 or 5 mL of the fresh medium containing 10 nM β-estradiol, supplemented with or without 5 mM NAM. Genomic DNA was extracted from the remaining culture using the GC prep method for qPCR.^62^ The same process was repeated every 1 to 3 days. The division number per day was calculated from the change of OD_620_.

#### Quantitative PCR (qPCR)

Genomic DNA was diluted ten times with distilled water before qPCR. Each qPCR solution (20 μL) contained 2 μL of diluted DNA, 10 μL of KOD SYBR qPCR Mix (TOYOBO), 0.04 μL of 50× ROX Reference Dye (TOYOBO), 2 pmol each of the forward and reverse primers. The primers used for qPCR are listed in Table S2. Each qPCR assay was performed in duplicate, using QuantStudio3 (Applied Biosystems) according to the manufacturer’s instructions. The amplification condition was initial denaturation at 98°C for 2 min followed by 40 times iteration of a 3-step thermal cycle composed of 98°C for 10 s, 55°C for 10 s, and 68°C for 30 s. All qPCR runs included 10-fold serial dilutions to generate standard curves. The quantity of *CUP1*, *ENA1, ymNG,* and *HIS3* was normalized to that of *ACT1*. The copy number of *CUP1*, *ENA1, ymNG,* and *HIS3* in the standard curves was calibrated by nanopore sequencing results in the BY4741 strain. The CNA/G for each gene was calculated with the below formula: CNA/G = (Copy number_Day T_ − Copy number_Day 0_)/Division number.

#### Modeling the contraction of extended *CUP1* array

To interpret the plot between the initial copy number and CNA/G (Figure 3H), we deduced a theoretical plot assuming that contraction occurs via homologous recombination between two *CUP1* repeat units, following second-order kinetics.

Let X be the copy number of repeat units, and k be the rate constant of homologous recombination between the repeat units. The rate equation is given by:

$$\frac{dX}{dt}=-kX^{2}$$

Let $X_{0}$ be the initial repeat unit copy number. Then, solving this differential equation yields:

$$X=\frac{X_{0}}{1+kX_{0}t}$$

The copy number as a function of time decreases along a rectangular hyperbola. Since CNA/G is defined as the difference in copy number at t=T and t=0 divided by the generation number G, it can be expressed as:

$$CNA/G=\frac{1}{G}\left(\frac{X_{0}}{1+kX_{0}T}-\frac{X_{0}}{1}\right)$$

Simplifying this:

$$CNA/G=\frac{1}{G}\left(\frac{-k{X_{0}}^{2}T}{1+kX_{0}T}\right)=-\frac{1}{G}\left(\frac{kT{X_{0}}^{2}}{1+kTX_{0}}\right)$$

Finally:

$$CNA/G=-\frac{1}{G}\left(\frac{{X_{0}}^{2}}{X_{0}+\frac{1}{kT}}\right)$$

If k and T are constants and $X_{0}$ is the variable, then CNA/G with respect to $X_{0}$ decreases along the sum of a linear function and a rectangular hyperbola. As the initial copy number $X_{0}$ increases, the contraction rate CNA/G asymptotically approaches a straight line, as observed in Figure 3H.

#### Nanopore sequencing

Genomic DNA was extracted using Monarch HMW DNA Extraction Kit for Tissue (NEB). We avoided vortexing to obtain high molecular weight DNA and used mixing by gentle pipetting with a wide-bore tip instead. DNA libraries for nanopore whole-genome sequencing were prepared using the ligation sequencing kit SQK-LSK109, SQK-LSK114 and the native barcoding kit EXP-NBD104, EXP-NBD114, or SQK-NBD114 (Oxford Nanopore Technologies) according to the manufacturer’s instructions. We modified the protocol of the ligation sequencing kit as follows: DNA fragmentation, omitted; duration of the enzymatic repair steps at 20°C and 65°C, both extended from 5 min to 30 min; and the duration of the ligation step, extended from 10 to 30 min; incubation time for elusion with 0.4× AMPure XP, extended from 10 min to 20 min. The library was sequenced with the flowcell FLO-MIN106D R9.4.1 using the MinION sequencer and FLO-PRO114M R10.4.1 using the PromethION 2 Solo sequencer (Oxford Nanopore Technologies). MinKNOW software was used to control the MinION and PromethION devices. The run time was set to 72 h. Base calling was performed using Guppy v6.5.7 and Dorado v0.7.3. The assessment of sequencing data was performed using NanoPlot.^49^

#### Dot plot analysis of nanopore reads

We used nanopore sequencing data in FASTA format to draw dot plots using YASS.^49^ We first selected reads spanning the entire array using 1-kb upstream and downstream sequences of the target array as queries of minialign (https://github.com/ocxtal/minialign) and then used these reads as the first input sequence for YASS. As the second input, we used the reference sequence of the repeat unit. By manually counting the diagonal lines in each dot plot, we determined the copy number of the repeat unit.

#### Translocation analysis using nanopore reads

We used nanopore sequencing data in FASTQ format, selected reads containing the *CUP1RU*, divided each read into 5′- and 3′-flanking regions of the *CUP1RU, and* extracted the flanking regions using Target_seq_extraction_single.py and Read_split_by_target.py (https://doi.org/10.5281/zenodo.11515698). We mapped reads to the S288c reference genome using Minimap2,^51^ SAMtools,^52^ and BEDtools.^53^ Data were visualized with the Integrative Genomics Viewer (IGV).^63^

#### Copy number estimation from nanopore reads

We used nanopore sequencing data in FASTQ format and mapped reads to the S288c reference genome (version R64-2-1, http://sgd-archive.yeastgenome.org/sequence/S288C_reference/genome_releases/S288C_reference_genome_R64-2-1_20150113.tgz) using SAMtools^52^ and BEDtools,^53^ and then normalized read count of each nucleotide was calculated using Bedgraph_norm_ratio.py (https://doi.org/10.5281/zenodo.11515696). Data were visualized with the IGV.^63^ To eliminate the effect of read clipping and achieve a more accurate estimation of repeat unit number, we collected all reads containing the repeat unit using minialign. We then used its reference sequence as a query in a BLAST^54^ search against the collected reads and estimated the copy number based on the number of BLAST hits.

#### Illumina sequencing

Genomic DNA was extracted using *Quick*-DNA Fungal/Bacterial Miniprep Kit (Zymo Research) and then fragmented to 300 bp using the S220 Focused-ultrasonicator (Covaris). DNA libraries with indexing were prepared using the ThruPLEX DNA-Seq kit (TaKaRa). We used the DNA Single Index Kit – 12S Set A or B (TaKaRa) for indexing according to the manufacturer’s instructions. The library was sequenced with MiSeq Reagent Kit v3 using the MiSeq instrument (Illumina). We mapped 2 × 75 bp reads to the S288c reference genome using Bowtie2,^55^ SAMtools,^52^ and BEDtools,^53^ and then normalized read count of each nucleotide was calculated using Bedgraph_norm_ratio.py (https://doi.org/10.5281/zenodo.11515696). Data were visualized with the IGV.^63^

#### Pulsed-field gel electrophoresis and Southern blot hybridization

Agarose-embedded yeast DNA was prepared using CHEF Genomic DNA Plug Kits (BioRad) according to the manufacturer’s instructions. DNA digested with or without EcoRI was subjected to 1% and 0.8% Certified Megabase Agarose (Bio-Rad) in 0.5× TBE and 1× TAE, respectively. Pulsed-field gel electrophoresis (PFGE) was performed using CHEF mapper XA (BioRad) according to the manufacturer’s instructions. PFGE running conditions were a 60–120 s pulse time, 120° angle, and 6 V/cm for 24 h at 14°C in a 1% agarose gel and 500 s pulse time, 106° angle, and 3 V/cm for 48 h at 14°C in a 0.8% agarose gel. The gel was then stained with SYBR Green I Nucleic Acid Gel Stain (Invitrogen) at room temperature for 30 min with shaking, destained in distilled water for 1 h, and the fluorescence signals were detected with ChemiDocTouch system (Bio-Rad). Transfer to the membrane was performed using Hybond-N+ (Cytiva) according to the manufacturer’s instructions. The blot was hybridized with a *CUP1* probe or outside probes at 55°C overnight after UV-crosslinking. The probe was generated by PCR using the primers listed in Table S2, followed by labeling with alkaline phosphatase using the labeling module of the AlkPhos Direct Labeling and Detection System kit (Cytiva). Following appropriate blot washing, chemiluminescent signals were generated using the CDP-Star Detection Reagent in the kit and detected with the ChemiDocTouch system (Bio-Rad). Images were processed with ImageJ software (National Institutes of Health).

#### Immunoblot analysis

The amount of histone H3 and acetylated histone H3 lysine 56 (H3K56ac) was analyzed by western blotting. Proteins were extracted as described previously,^64^ and separated with sodium dodecyl sulfate-polyacrylamide gel electrophoresis using 12% Mini-PROTEAN TGX Precast Protein Gel (Bio-Rad). Transfer to the membrane was performed with iBind Western System (Thermo Fisher Scientific) according to the manufacturer’s protocol. Primary antibodies to detect histone H3 and H3K56ac were Anti-Histone H3 Antibody, CT, pan, clone A3S, rabbit monoclonal antibody (1:500, Sigma-Aldrich), and Histone H3K56ac rabbit polyclonal antibody (1:2500, Active Motif), respectively. The secondary antibody was Anti-rabbit IgG, HRP-linked Antibody (1:2000, Cell Signaling Technology). Following incubation with Clarity Western ECL Substrate (Bio-Rad), chemiluminescent signals were detected with the ChemiDocTouch system (Bio-Rad). Gel images were processed with ImageJ software.

#### Fluorescence microscopy and image processing for yeast cells

Image acquisitions of yeast cells were performed on a microscope (Ti-E, Nikon, Tokyo, Japan) with a 20× objective lens (CFI Plan Apo Lambda 20X, MRD00205, Nikon), a sCMOS camera (ORCA-Fusion BT, C15440-20UP, Hamamatsu photonics, Hamamatsu, Japan), and a solid-state illumination light source (SOLA SE II, Lumencor, Beaverton, OR, USA). Image acquisition was controlled by NIS-Elements version 5.3 (Nikon). Z-stacks were 7 × 0.9 μm. For imaging of mNeonGreen, a filter set (LED-YFP-A, Semrock, Rochester, NY, USA) was used with excitation light power set at 7% and the exposure time set at 200 msec/frame. For imaging of mCherry, a filter set (LED-TRITC-A, Semrock) was used with excitation light power set at 30% and exposure time set at 300 msec/frame. For imaging of miRFP682, a filter set (LED-Cy5.5-A, Semrock) was used with excitation light power set at 10% and exposure time set at 200 msec/frame. For imaging of TagBFP, a filter set (LED-DAPI-A, Semrock) was used with excitation light power set at 50% and exposure time set at 700 msec/frame. For DIC (differential interference contrast) image acquisition, the exposure time was set at 50 ms/frame. DIC images were captured only at the middle position of the Z-stacks.

Image processing and analysis were performed using Fiji.^65^ To generate 2-dimensional images of fluorescence channels from Z-stacks, background subtraction (sliding paraboloid radius set at 5 pixels with disabled smoothing) and maximum projection using 7 Z-slices were performed. Maximum projected fluorescence images and corresponding smoothed DIC images were superimposed. After global adjusting of brightness and contrast and cropping of the images, sequences of representative images were generated.

#### BITREx of *EGFP* reconstitution reporter in HEK293T cells

Human embryonic kidney 293T cell (HEK293T) cells were cultured DMEM (Gibco) supplemented with 10% fetal bovine serum (Gibco) and 1% penicillin–streptomycin (Gibco). Cells were maintained at 37°C in a humidified atmosphere containing 5% CO_2_.

The donor vector mCherry-PuroR-FP-SV40 ori-EGF-gRNA1ts was derived from the plasmid pyChrIV545kb_FP-SV40ori-EGF-yChrIV592kb_RFP_puroR.^35^ We also constructed the nCas9-gRNA1 expression plasmid by inserting the gRNA1-coding sequence into the AIO-Puro plasmid.^48^ Using the Lipofectamine 3000 reagent (Invitrogen), we co-transfected the donor plasmid, the donor cleavage vector (VKG1-gRNA-pX330),^32^ and the locus-specific cleavage vector (AAVS1 T2 CRISPR in pX330)^47^ into HEK293T cells to facilitate knock-in of the donor plasmid at the target locus via the VIKING method.^32^

Puromycin (0.3 μg/mL) was added to the culture at 24 h after transfection. Following 48 h of cultivation, the cells were transfected with the nCas9-gRNA1 expression plasmid. Puromycin (2.0 μg/mL) was then added to the culture at 24 h after the second transfection, and the cells were grown for an additional 72 h.

We designed a primer pair spanning the cleavage site of the donor plasmid to specifically detect residual donor plasmids while excluding signals from those integrated into the genome. The copy number of residual donor plasmid was estimated from the qPCR results, considering the hypotriploid nature of HEK293 cells.^66^

#### Flow cytometric analysis of transfected HEK293T cells

Transfected HEK293T cells with the *EGFP* reconstitution reporter were washed twice with phosphate-buffered saline (PBS) and detached from the dishes using 0.25% trypsin-EDTA (Gibco). The cells were then resuspended in PBS containing 0.2% bovine serum albumin (Thermo Fisher Scientific) and filtered through a 50 μm nylon mesh to obtain a single-cell suspension. Cell density was adjusted to 5 × 10^6^ cells/mL. Cells were analyzed using a BD FACSAria Fusion cell sorter (BD Biosciences). Data acquisition was performed using BD FACSDiva software (BD Biosciences). At least 10,000 events were collected per sample. Data were analyzed using FlowJo software (BD Biosciences), and gates were set based on isotype controls.

#### Flow sorting of EGFP-positive HEK293T cells

EGFP and mCherry-positive cells were sorted using a BD FACSAria Fusion cell sorter (BD Biosciences). The excitation wavelength for EGFP was set to 488 nm, and EGFP florescence was detected using a 530/30 nm bandpass filter. The excitation wavelength for mCherry was set to 561 nm, and mCherry fluorescence was detected using a 610/20 nm bandpass filter. Untransfected HEK293T cells were used as negative controls to set the gates for GFP and mCherry-positive cells. At least 10,000 events were collected per sample.

#### Fluorescence microscopy and image processing for HEK293T cells

Image acquisitions of HEK293T cells were performed on an imaging system (EVOS M7000, Thermo Fisher Scientific) with a 20× objective lens (NA 0.70, Olympus, AMEP4765EO). For imaging of EGFP, a filter set (EVOS Light Cube GFP 2.0, Thermo Fisher Scientific, AMEP4951) was used with the exposure time set at 2 msec/frame. For imaging of mCherry, a filter set (EVOS Light Cube Texas Red 2.0, Thermo Fisher Scientific, AMEP4955) was used with the exposure time set at 50 msec/frame. For bright field image acquisition, the exposure time was set at 10 ms/frame. Image processing and analysis were performed using Fiji.^65^ Background subtraction (sliding paraboloid radius set at 5 pixels with disabled smoothing) were performed. Fluorescence images and corresponding bright field images were superimposed. After global adjusting of brightness and contrast and cropping of the images, sequences of representative images were generated.

#### BITREx of the D4Z4 array in HEK293T cells

We constructed nCas9-gRNA expression plasmids by inserting the coding sequences for gRNAh1, gRNAh2, gRNAh3, and gRNAh4 into the AIO-Puro plasmid.^48^ Each plasmid was transfected into HEK293T cells following the procedure described above.

#### Nanopore sequencing of genomic DNA prepared from HEK293T cells

Genomic DNA was extracted from HEK293T cells using NucleoSpin Tissue (Macherey-Nagel) and Monarch HMW DNA Extraction Kit for Cells & Blood (Monarch) according to the manufacturer’s protocol. The extracted DNA was then used for ligation-based nanopore whole-genome sequencing library preparation. Reads containing the *EGFP* reconstitution reporter were identified using minialign (https://github.com/ocxtal/minialign) and subsequently analyzed to generate dot plots with YASS.^50^ Minimap2^51^ was used to identify reads containing a unique sequence in the D4Z4 repeat unit (T2T-CHM13v2.0, chr.4:193,434,263–193,435,217) and reads containing a unique region flanking the D4Z4 array (T2T-CHM13v2.0, chr.4:193,427,855–193,437,559). These reads were then used to estimate the D4Z4 copy number, as described above.

### Quantification and statistical analysis

One-way ANOVA test and Student’s t test were employed to calculate *p* values, as indicated in the figure legends. In general, results were considered statistically significant when *p* < 0.05.

## Supplemental information

Document S1. Figures S1–S9 and Table S1

Table S2. Yeast strains used in this study, related to STAR Methods and Figures 1–5 and S2–S8

Table S3. PCR primers used in this study, related to STAR Methods and Figures 1–6 and S2–S9

Table S4. Plasmids used in this study, related to STAR Methods and Figures 1–6 and S2–S9

Table S5. gRNAs and crRNAs used in this study, related to STAR Methods and Figures 1–6 and S2–S9

Document S2. Article plus supplemental information
