# Supplementary material for: Strategic targeting of Cas9 nickase expands tandem gene arrays
Source: Cell Genom. 2025 Mar 20;5(4):100811. doi: 10.1016/j.xgen.2025.100811 (PMC12008805; doi:10.1016/j.xgen.2025.100811)
Supplement: Document S1. Figures S1–S9 and Table S1 [file mmc1.pdf]

Cell Genomics, Volume *5*

Supplemental information

Strategic targeting of Cas9 nickase expands tandem gene arrays

Hiroaki Takesue, Satoshi Okada, Goro Doi, Yuki Sugiyama, Emiko Kusumoto, and Takashi Ito

Table S1: CNA/G values for six two-unit arrays at three loci, related to Figures 1, 4, S7, and S8

Array type	Interrupting sequence	Repeat unit length (bp)	CNA/G at each locus (top: Day 0 to 3; bottom: Day 3 to10)		
			CUP1 (chr VIII)	*HO* (chr IV)	*X-2* (chr X)
2×CUP1RU	none	1,988	0.153<br>0.216	–	–
	HIS3<br>4 fluorescent protein genes<br>plasmid backbone	10,339	0.102<br>0.004	0.038<br>0.003	0.051<br>0.008
	HIS3<br>4 fluorescent protein genes<br>plasmid backbone<br>*ARS305*	10,490	0.110<br>0.141	0.043<br>0.024	0.032<br>0.059
2×ymNGRU	none	1,642	0.246<br>0.237	–	–
	HIS3<br>plasmid backbone	4,994	0.015<br>0.050	0.039<br>0.068	0.197<br>0.001
	HIS3<br>plasmid backbone<br>*ARS305*	5,072	0.069<br>0.087	0.038<br>0.053	0.120<br>0.144
14×CUP1RU	none	1,988	0.492<br>0.577	–	–

Note:
The six two-unit arrays were integrated at three genomic loci and subjected to BITREx using gRNA1. CNA/G values were calculated from qPCR data at days 0, 3, and 10 for each indicated two-unit array. The values from day 0 to 3 and day 3 to 10 are shown in the top and bottom lines of each box, respectively. A minus sign indicates that the corresponding strain was not available. As a control, CNA/G values for native *CUP1* array at *CUP1* locus are also included. Notably, arrays embedded at the same locus displayed varying efficiencies, underscoring the importance of repeat unit characteristics, such as sequence composition, length, the presence or absence of an ARS, and the inclusion of intervening sequences. The presence of an internal ARS contributes to maintaining the CNA/G value after day 3. However, during the early stages of BITREx, when array lengths are relatively short, this effect can be overridden by other sequence features. For instance, at the *CUP1* locus, the uninterrupted *2×ymNGRU* array outperformed the uninterrupted *2×CUP1RU* array from day 0 to day 3, although the former lacks an internal ARS while the latter contains one (*ARS810/811*). This difference diminished between days 3 and 10, likely because the initial advantage of *2×ymNGRU*, attributed to its sequence composition, was progressively offset by the influence of the internal ARS in *2×CUP1RU*. Moreover, identical arrays demonstrated varying behaviors depending on the locus in which they were embedded, highlighting the impact of the local environment, including the distance from nearby ARS, epigenetic status, and so on. For example, the *2×ymNGRU* array interrupted by *HIS3* exhibited a marked CNA/G decline after day 3 at the *X-2* locus but not at the *CUP1* or *HO* loci.

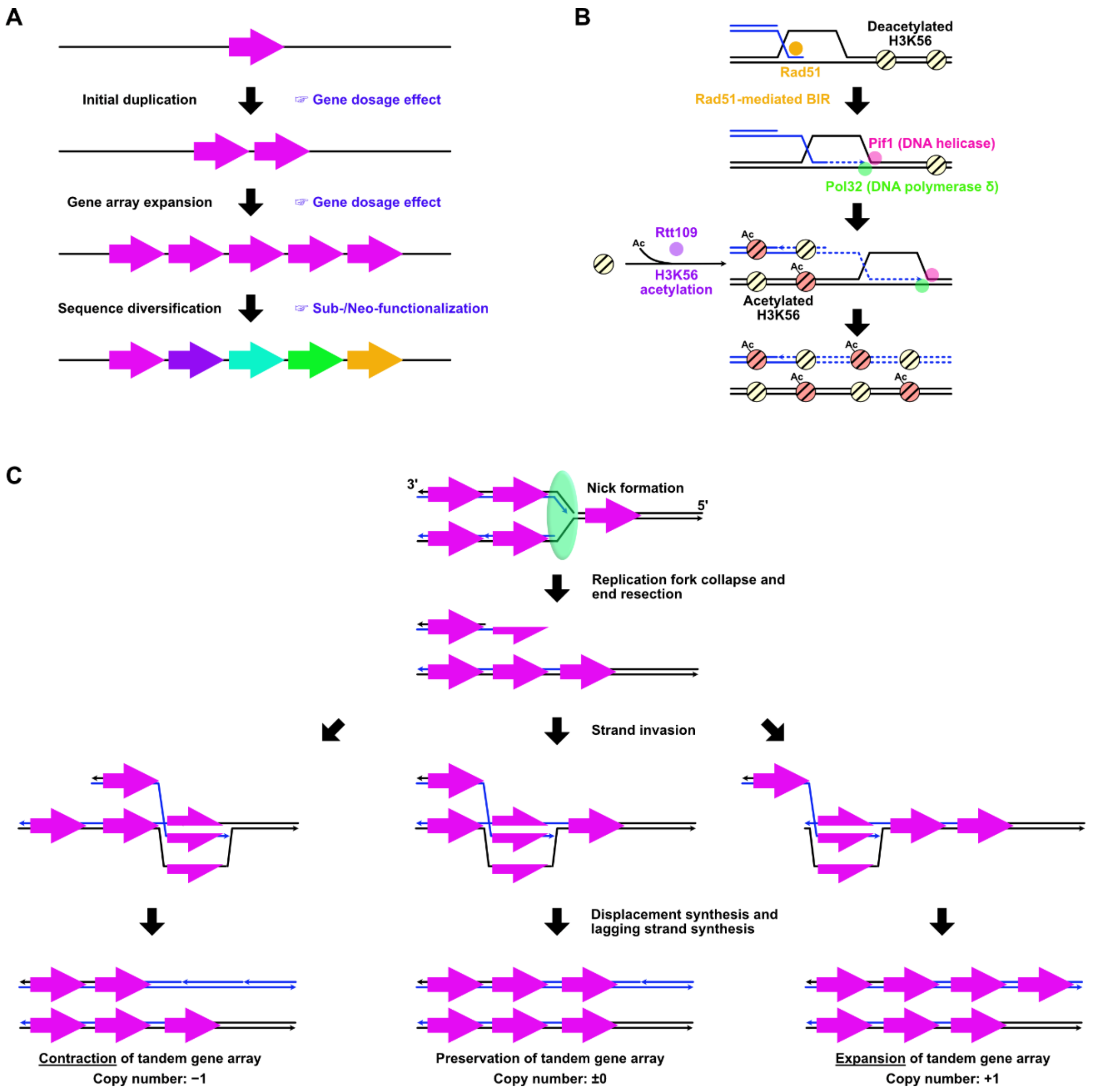

Figure S1: Conceptual foundations of BITREx, related to Figure 1

(A) Roles of gene duplication and tandem gene array expansion in adaptation and evolution. Initial duplication and subsequent gene array expansion exert gene dosage effect. Sequence diversification leads to sub-/neo-functionalization. See text for detail.

(B) Mechanism of BIR.[10,11] The initial step of BIR is the invasion of ssDNA generated by end resection at seDSB into the donor sequence. Rad51 mediates this step in most BIR events. The invaded ssDNA primes the displacement synthesis catalyzed by DNA polymerase δ (Pol δ). Importantly, this step is dependent on Pol32, a Pol δ subunit that is not required for normal S-phase replication. Another essential gene for BIR is PIF1, which encodes the DNA helicase indispensable for BIR fork (D-loop) progression. In addition, BIR is suppressed by Rtt109-catalyzed acetylation at Lys-56 of histone H3 (H3K56ac). The yeast proteins Rad51, Pol32, and Pif1 have mammalian homologs—RAD51, POLD3, and PIF1, respectively. In contrast, Rtt109 is a fungus-specific histone acetylase with no mammalian homolog.

(C) Outcomes of BIR initiated from an internal unit of a tandem gene array.

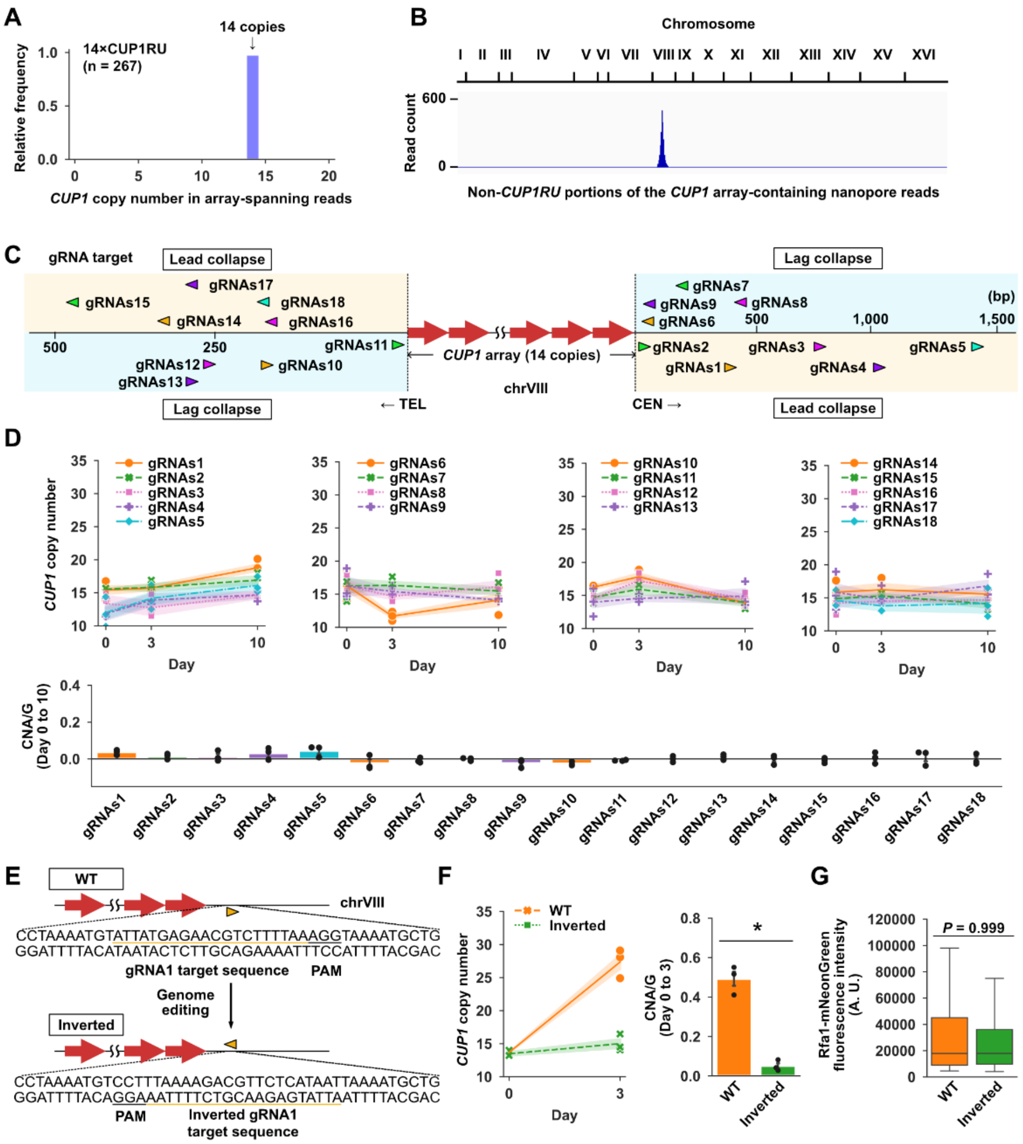

Figure S2: BITREx of *CUP1* array, related to Figure 1

(A) *CUP1* array length of the parental strain used in this study. Nanopore reads containing both the 5′- and 3′-flanking regions of *CUP1* array were used to determine the distribution of *CUP1RU* copy number.

(B) Genomic location of *CUP1* array-flanking sequences in the parental strain used in this study. Nanopore reads containing *CUP1RU* were selected and their non-*CUP1RU* portions were mapped to the reference genome sequence.

(C) *CUP1* array and target sites of ineffective gRNAs (gRNAs1–gRNAs18). Similar to Figure 1C.

(D) Performance of 18 gRNAs shown in (C). Similar to Figures 1D and 1E. Shading and error bar, SD (n = 3 biological replicates).

(E) Inversion of the gRNA1 target site. In this strain, gRNA1 should make Cas9^{D10A} induce a nick on the lagging strand template.

(F) BITREx in the wild-type (WT) and inverted strains. Left, CNA of *CUP1*; right, CNA/G. Shading and error bar, SD (n = 3 biological replicates).

(G) Box plots showing Rfa1-mNeonGreen fluorescence in the WT and inverted strains with Cas9 and gRNA1. Since Rfa1 accumulates on ssDNA generated by end-resection at DSB sites as a component of the RPA complex, the fluorescence intensity serves as an indicator of gRNA1-guided Cas9 cleavage.

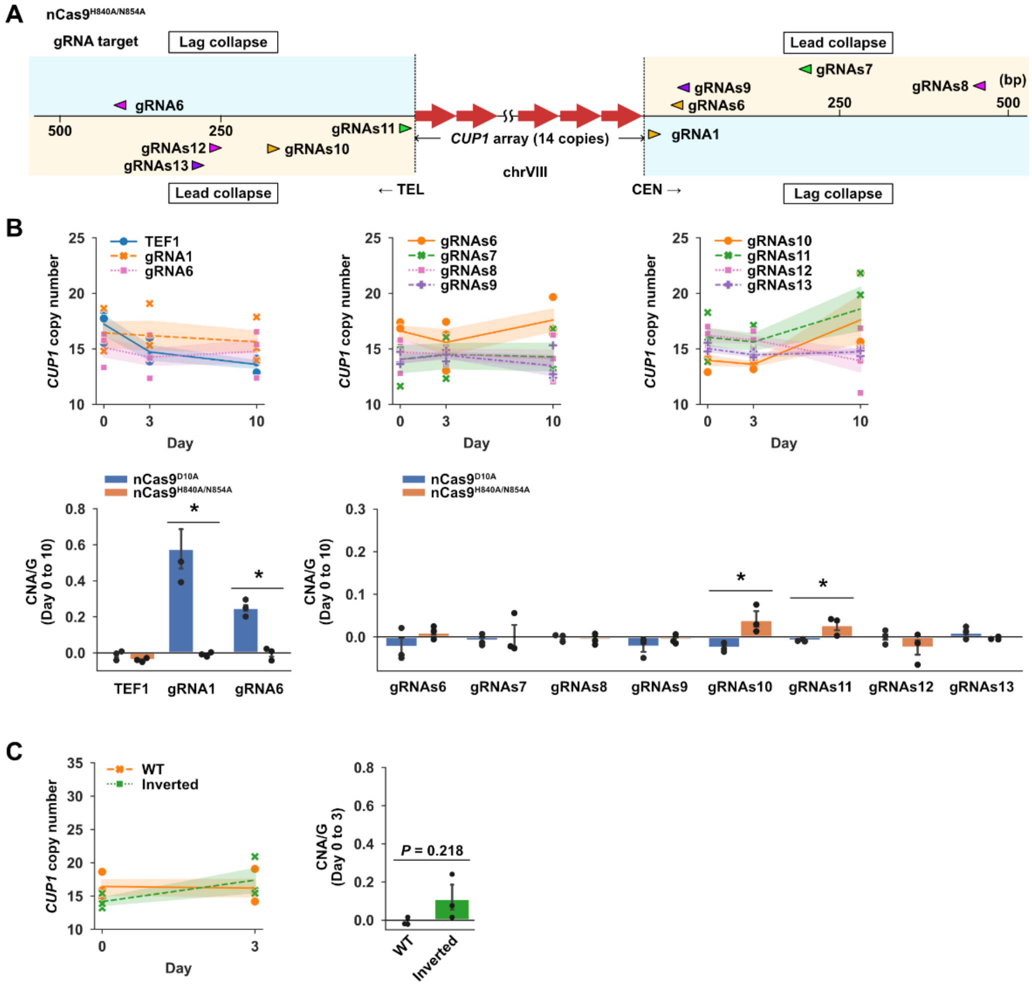

Figure S3: Requirement of lead collapse for BITREx, related to Figure 1

(A) *CUP1* array and target sites of 10 gRNAs used with nCas9$^{H840A/N854A}$. Two gRNAs (gRNA1 and gRNA6) were effective when used with nCas9^{D10A} and should induce lag collapse when used with nCas9$^{H840A/N854A}$. The remaining eight gRNAs (gRNAs6–gRNAs13) were ineffective when used with nCas9^{D10A} (Figure S2D) and should induce lead collapse when used with nCas9$^{H840A/N854A}$. Note that Cas9$^{H840A/N854A}$ has been reported to surpass Cas9^{H840A} in terms of correct nick formation frequency and reduced occurrence of unwanted indels.[15]

(B) BITREx using nCas9$^{H840A/N854A}$. Similar to Figures 1D and 1E. Shading and error bar, SD (n = 3 biological replicates). *P < 0.05 (Student's t-test). Notably, when combined with Cas9$^{H840A/N854A}$, the two gRNAs that performed most efficiently with Cas9^{D10A} (gRNA1 and gRNA6) failed to increase *CUP1* copy number. Conversely, two gRNAs that were ineffective with Cas9^{D10A} (gRNAs10 and gRNAs11) induced a small but significant *CUP1* CNA.

(C) BITREx using gRNA1 and nCas9$^{H840A/N854A}$ in the inverted strain (Figure S2E). Shading and error bar, SD (n = 3 biological replicates). Note that Cas9$^{H840A/N854A}$ with gRNA1 induced a weak increase in the copy number in the inverted strain.

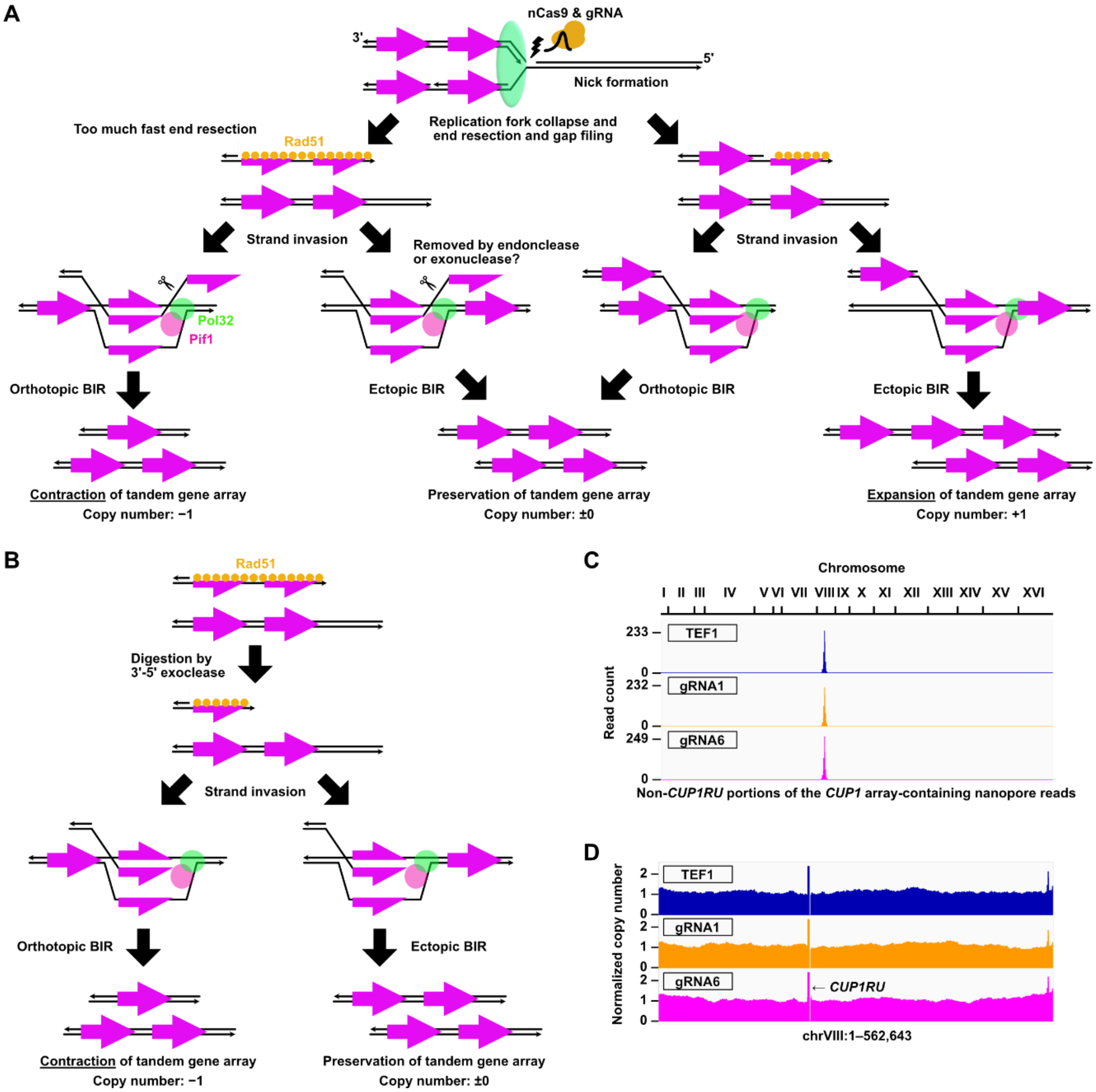

(legend on next page)

Figure S4: Array contraction, gross rearrangement, and target site mutation during BITREx, related to Figure 1

(A) Array contraction by extensive 5′-to-3′ end resection. If extensive 5′-to-3′ end-resection converts not only the terminal but also the second terminal repeat unit to ssDNA, the latter ssDNA may hybridize with the terminal repeat unit on the sister chromatid. In this scenario, the terminal repeat unit of the invading ssDNA strand is left behind as a flap. If this flap is degraded by flap endonucleases such as Rad1-Rad10, the invading ssDNA (i.e., the second terminal repeat unit) can initiate BIR, leading to the contraction of the tandem array.

(B) Array contraction by extensive 3′-to-5′ end resection. A similar situation to (A) could occur if the 3′-to-5′ exonuclease activity of Pol δ excessively degrades or "chews backs" the invading strand.[41]

(C) Genomic location of *CUP1* array-flanking sequence in the strains subjected to BITREx with the indicated gRNAs for 3 days. Similar to Figure S2B. We collected all *CUP1RU*-containing reads and mapped their non-*CUP1RU* sequences to the reference genome. As expected, almost all of them were derived from the *CUP1* array-flanking regions on chromosome VIII. Each of the few abnormal junctions was unique and appeared only once, likely representing artifacts or chimeric molecules generated during ligation-based library preparation: true translocation junctions, in contrast, would be expected to appear in multiple independent reads, given the >200-fold genome coverage achieved.

(D) Normalized read counts across chromosome VIII in nanopore sequencing. Read counts were normalized to the average counts of genomic regions excluding rRNA, *CUP1RU*, Ty elements, and mitochondrial DNA. Note that while the sacCer3 reference genome sequence contains two copies of *CUP1RU*, the second copy is masked with 'N' prior to mapping. Consequently, the normalized read count directly reflects the *CUP1RU* copy number. The gap in read counts adjacent to *CUP1RU* is due to the masking. The copy numbers of *CUP1RU* were estimated to be 17.5, 30.5, and 20.4 in cells subjected to 3-day BITREx using *TEF1* gRNA, gRNA1, and gRNA6, respectively. The average read depth of chromosome VIII was comparable to the genome-wide average, except for the *CUP1* locus and the polymorphic subtelomeric regions.

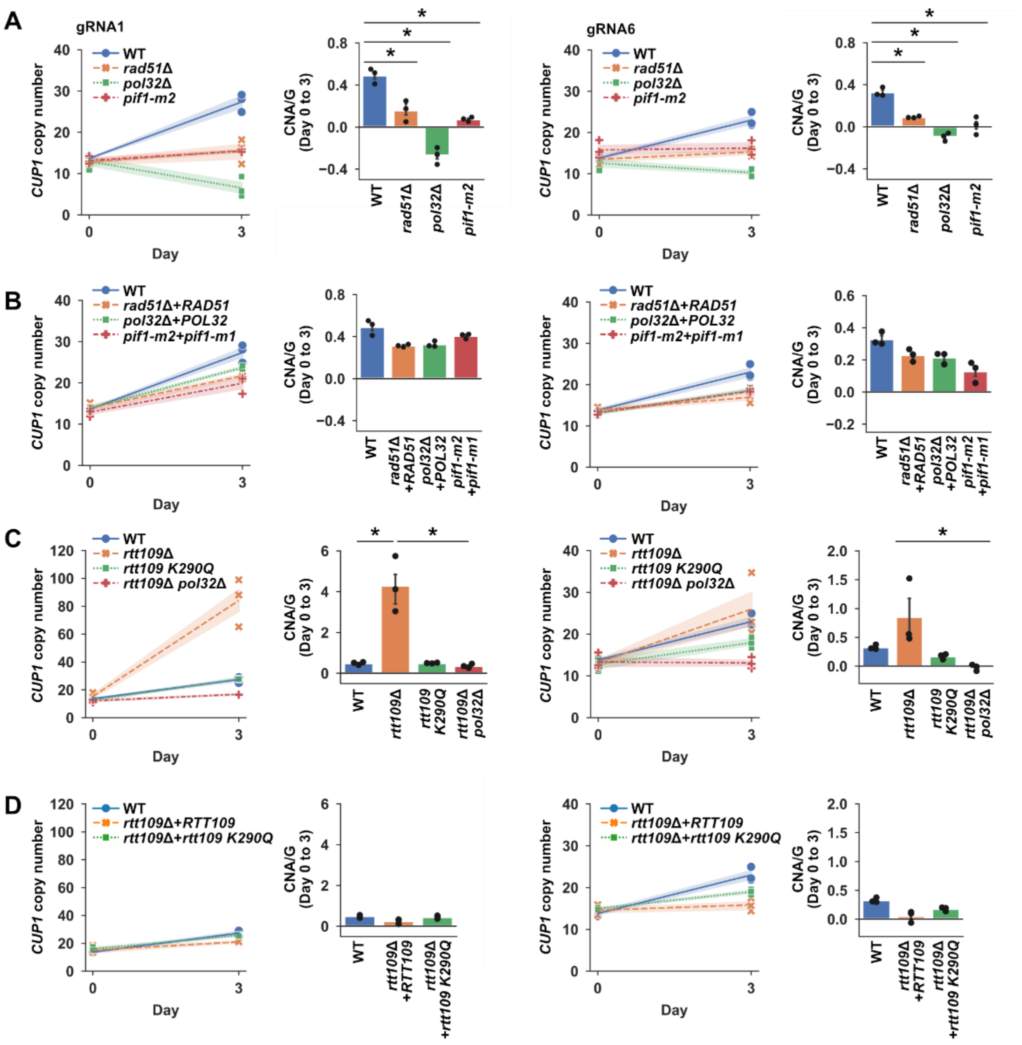

Figure S5: Genetic evidence for BIR to mediate BITREx, related to Figure 1

(A) BITREx defects in *rad51*Δ, *pol32*Δ, and *pif1-m2* cells. Note that *PIF1* contains two initiation Met codons, with the first and second codons directing the synthesis of its mitochondrial and nuclear isoforms, respectively.[17] The *pif1-m2* allele substitutes the second initiation Met codon with an Ala codon, thereby restricting synthesis to the mitochondrial isoform only.[17] The *CUP1* copy number was measured by qPCR on days 0 and 3 to calculate CNA/G in each mutant. Two gRNAs (gRNA1 and gRNA6) were used. Shading and error bar, SD (n = 3 biological replicates). *P < 0.05 (Student's t-test).

(B) Suppression of BITREx defects in *rad51*Δ, *pol32*Δ, and *pif1-m2* cells by episomal copies of *RAD51*, *POL32*, and *pif1-m1*, respectively. The *pif1-m1* allele substitutes the first initiation Met codon with an Ala codon, thereby restricting synthesis to the nuclear isoform only.[17] Shading and error bar, SD (n = 3 biological replicates). *P < 0.05 (Student's t-test).

(C) Enhancement of BITREx in *rtt109*Δ cells but not in *rtt109 K290Q* and *rtt109*Δ *pol32*Δ cells. Rtt109 is the sole enzyme responsible for H3K56ac, but it also contributes to H3K9ac.[18] While no amino acid substitution was known to selectively abolish the H3K56 acetylase activity, K290Q substitution selectively abolishes the H3K9 acetylase activity. Shading and error bar, SD (n = 3 biological replicates). *P < 0.05 (Student's t-test).

(D) Suppression of BITREx enhanced in rtt109Δ cells by episomal copies of *RTT109* and *rtt109 K290Q*. Shading and error bar, SD (n = 3 biological replicates). *P < 0.05 (Student's t-test).

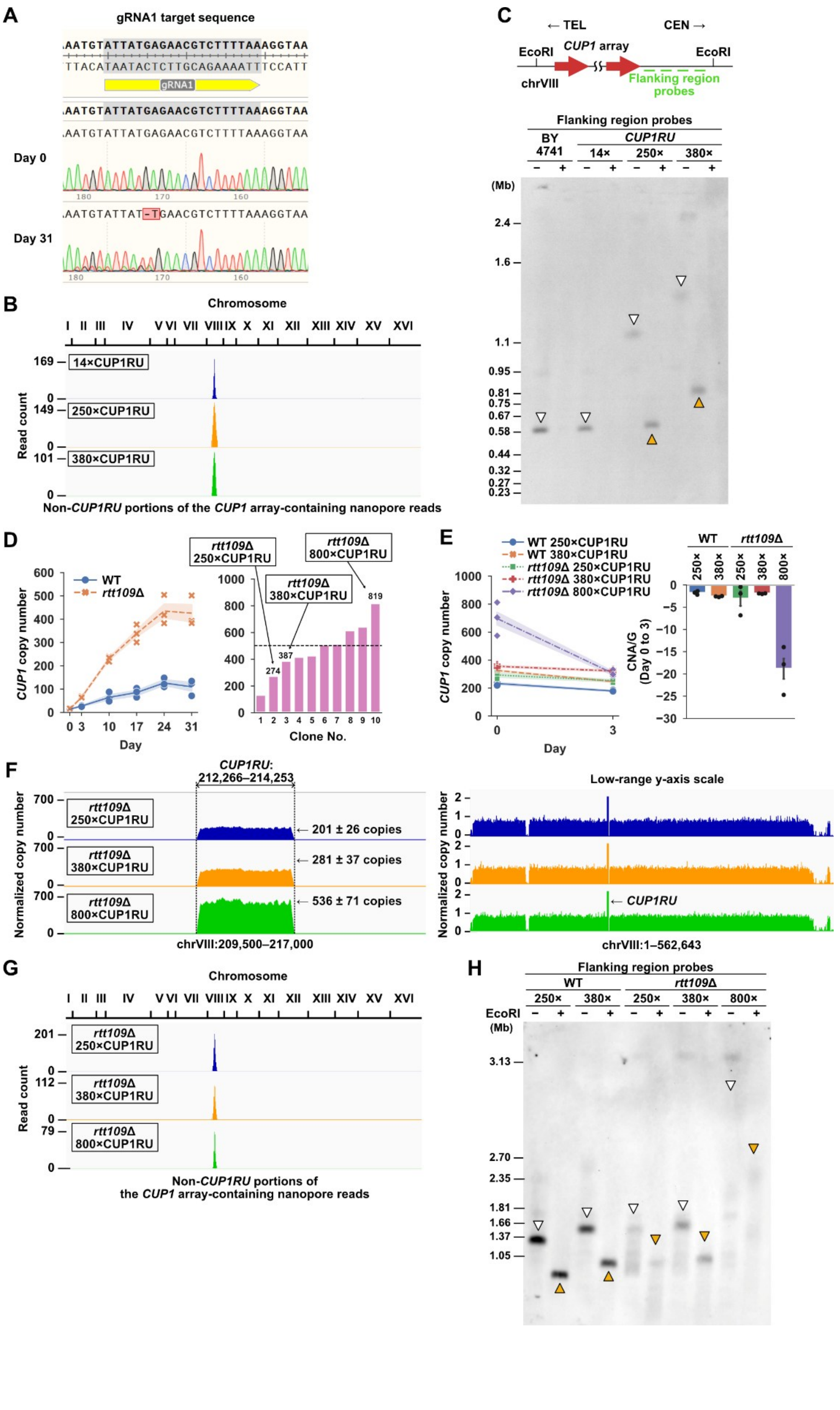

(legend on next page)

Figure S6: Long-term BITREx, related to Figure 2

(A) Mutation at the gRNA1 target site. Electropherograms are shown for Sanger sequencing of a PCR amplicon including the gRNA1 target site obtained from a cell population exhibiting a notable decline in copy number between days 24 and 31 (Figure 2A).

(B) Genomic location of *CUP1* array-flanking sequences in the strains subjected to long-term BITREx. Similar to Figure S2B.

(C) PFGE analysis of *CUP1* arrays expanded by long-term BITREx in wild-type cells. Similar to Figure 2H, but Southern blot hybridization was performed using the flanking region probes shown in the upper panel.

(D) CNA of *CUP1* over the 31-day BITREx with gRNA1 in *rtt109*Δ cells. Shading, SD (n = 3 biological replicates). *CUP1* copy numbers of 10 randomly picked clones on day 31 are shown in the right panel. The dashed line indicates the average copy number, which is 502.

(E) Stability of extended *CUP1* arrays. Each strain was cultivated for 3 days without BITREx induction. Shading and error bar, SD (n = 3 biological replicates).

(F) Normalized read counts in Illumina sequencing of the *rtt109*Δ strains subjected to long-term BITREx. Similar to Figure 2F.

(G) Genomic location of *CUP1* array-flanking sequences in the *rtt109*Δ strains subjected to long-term BITREx. Similar to Figure S2B.

(H) PFGE analysis of *CUP1* arrays expanded by long-term BITREx in wild-type and *rtt109*Δ cells. Similar to Figure 2J, but flanking region probes were used for Southern blot hybridization. White and orange arrowheads indicate chromosome VIII and EcoRI-excised *CUP1* array, respectively.

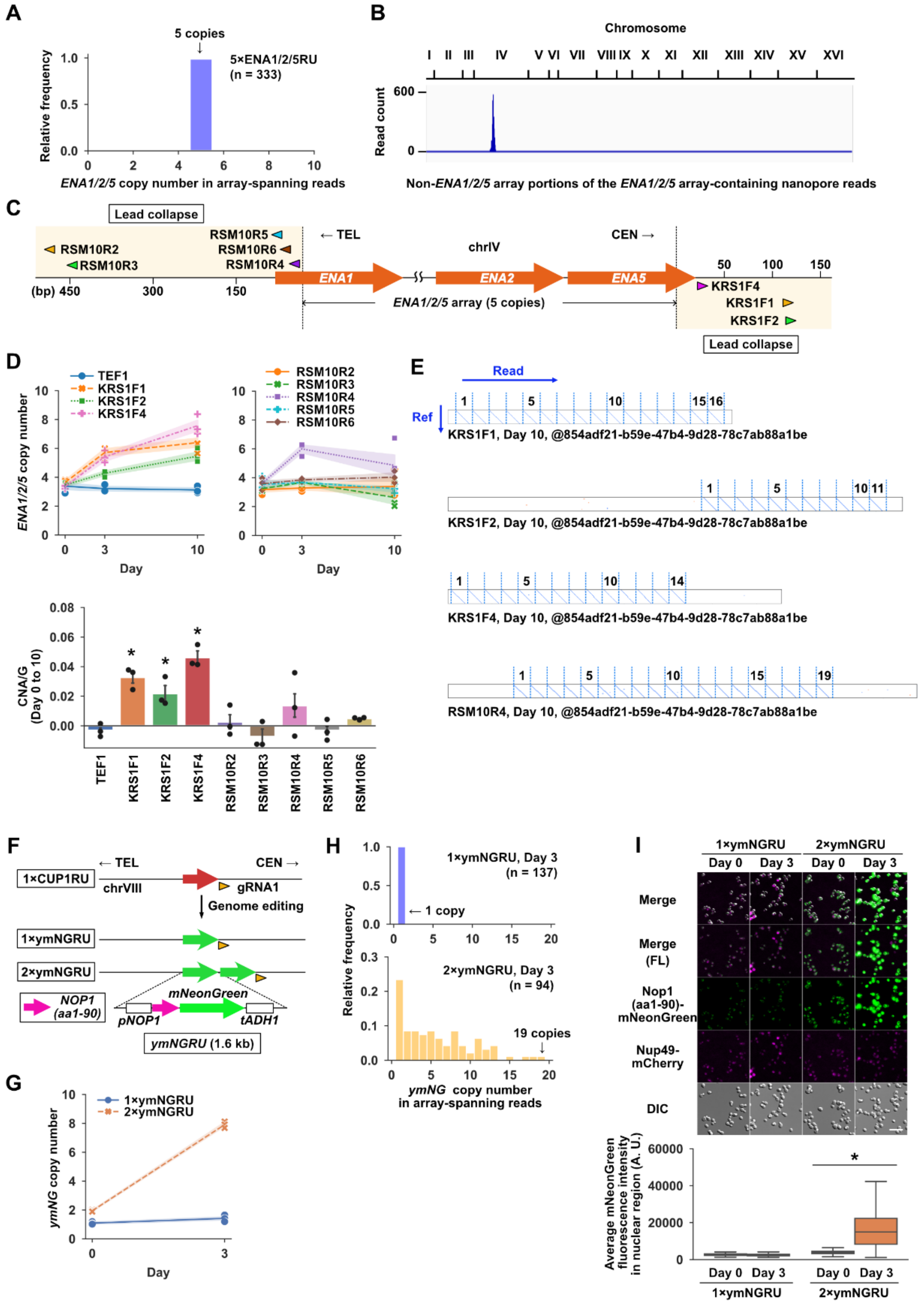

(legend on next page)

Figure S7: BITREx of non-*CUP1* arrays, related to Figure 4

(A) *ENA1/2/5* array length of the parental strain used in this study. The *ENA1/2/5* array comprises a tandem array of three paralogous genes, namely *ENA1*, *ENA2*, and *ENA5*, on chromosome IV in the S288C reference genome sequence. However, other strains were reported to have four or more paralogs,[25,26] and the strain used in this study has five paralogs. Nanopore reads containing both the 5′- and 3′-flanking regions of *ENA1/2/5* array were used to determine the distribution of *ENA1/2/5* copy number.

(B) Genomic location of *ENA1/2/5* array. Nanopore reads containing *ENA1/2/5* were selected, and their non-*ENA1/2/5* portions were mapped to the reference genome sequence.

(C) *ENA1/2/5* array and target sites of gRNAs tested in this study. Similar to Figure 1C.

(D) Performance of eight gRNAs shown in (C). Similar to Figures 1D and 1E. Shading and error bar, SD (n = 3 biological replicates). The population average copy number increased to as many as 8.4 copies after 10 days of BITREx.

(E) Representative dot plots comparing nanopore reads to the reference sequence of *ENA1/2/5* repeat unit. Genomic DNAs prepared from the cells with four gRNAs (KRS1F1, KRS1F2, KRS1F4, or RSM10R4) on day 10 were used for the nanopore sequencing.

(F) *ymNGRU* arrays generated on chromosome VIII. A single *CUP1RU* at the *CUP1* locus on chromosome VIII was replaced by a single copy or tandemly duplicated copies of *ymNGRU* using genome editing. The *ymNGRU* consists of a yeast codon-optimized coding sequence for the fluorescent protein mNeonGreen (*ymNG*), preceded by the *NOP1* promoter and the coding sequence for the nuclear localization signal-containing domain of Nop1 (amino acid residues 1–90),[27] and followed by the *ADH1* terminator.

(G) CNA of *ymNG* by BITREx with gRNA1 in the strain bearing either a single copy *ymNGRU* (1×ymNGRU) or a two-unit *ymNGRU* array (2×ymNGRU). Shading, SD (n = 3 biological replicates). The population average copy number increased to as many as 8.1 copies after 3 days of BITREx.

(H) Distribution of *ymNGRU* copy number in nanopore reads spanning the entire array in the 1×ymNGRU and 2×ymNGRU strains on day 3 of BITREx.

(I) Fluorescence microscopic analysis of 1×ymNGRU and 2×ymNGRU strains. Upper panel, representative microscopic images. These strains have *NUP49-mCherry* to visualize the nuclei (magenta). FL, fluorescence; DIC, differential interference contrast. Scale bar, 20 μm. Lower panel, quantification of fluorescence intensity. Box plots indicate the distribution of the average mNeonGreen fluorescence intensity in the nuclear region. The bottom and top of the box show the first and third quartiles, respectively. The bar in each box represents the median value, and the error bars represent the range of values. *P < 0.001 (one-way ANOVA test).

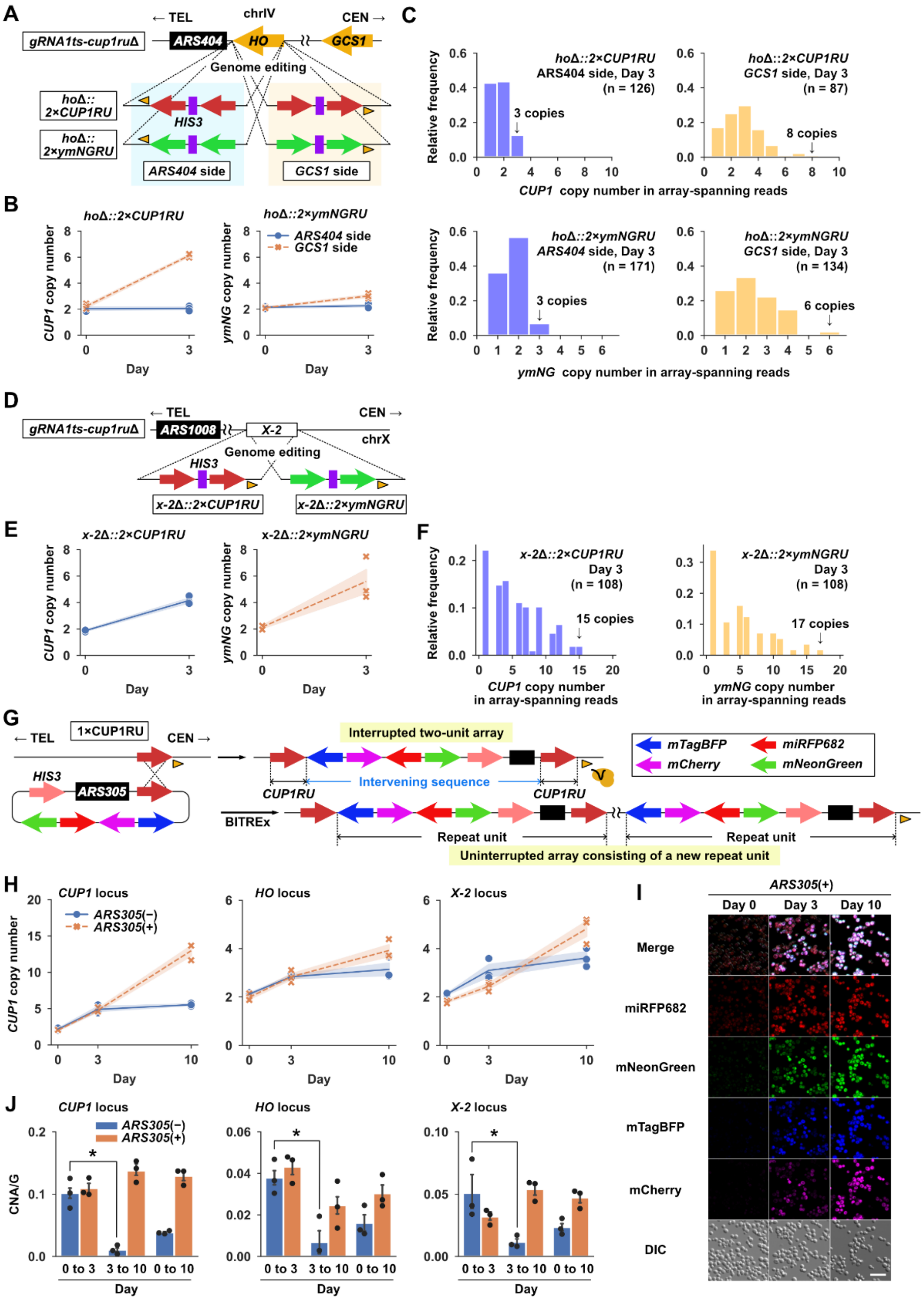

(legend on next page)

Figure S8: BITREx of interrupted two-unit arrays, related to Figure 4

(A) Interrupted two-unit arrays of *CUP1RU* and *ymNGRU* inserted at the *HO* locus on chromosome IV. Note that these arrays include *HIS3* between the two repeat units to facilitate strain construction. They can thus be interpreted as interrupted two-unit arrays of *CUP1RU/ymNGRU*. We inserted the interrupted two-unit array in two orientations: in one strain, the nick can be introduced on the ARS404-proximal side, and in the other strain, on the *GCS1*-proximal side of the array. Orange arrowheads, gRNA1 target site.

(B) CNA of *CUP1* and *ymNG* in the *ho*Δ*::2×CUP1RU* and *ho*Δ*::2×ymNGRU* strains, respectively. Shading, SD (n = 3 biological replicates).

(C) Distribution of *CUP1/ymNG* copy number in nanopore reads spanning the entire array obtained on day 3.

(D) Interrupted two-unit arrays of *CUP1RU* and *ymNGRU* integrated to the *X-2* locus[29] on chromosome X. *ARS1008* is the nearest ARS in the side opposite to the gRNA1 target site (orange arrowhead).

(E) CNA of *CUP1* and *ymNG* in the *x-2*Δ*::2×CUP1RU* and *x-2*Δ*::2×ymNGRU* strains, respectively. Shading, SD (n = 3 biological replicates).

(F) Distribution of *CUP1/ymNG* copy number in nanopore reads spanning the entire array obtained on day 3.

(G) BITREx of a two-unit *CUP1RU* array interrupted by an intervening sequence containing four fluorescent protein genes (*mTagBFP*, *miRFP682*, *mCherry*, and *mNeonGreen)* and *HIS3*. This array was generated through recombination between a genomic single-copy *CUP1RU* and a plasmid bearing a *CUP1RU* with the intervening sequence. The first cycle of BIR at the interrupted 2×*CUP1RU* array generates an uninterrupted two-unit array consisting of a new repeat unit that includes the intervening sequence and *CUP1RU*.

(H) CNA of *CUP1* in the strains bearing the interrupted *CUP1* array in (G) at *CUP1*, *HO*, or *X-2* loci. Isogenic stains without the embedded *ARS305* were also shown. Shading, SD (n = 3 biological replicates). *P < 0.05 (Student's t-test). Notably, the effects of the embedded *ARS305* were evident between days 3 and 10 but not between days 0 and 3.

(I) Microscopic images of the strain bearing the interrupted *CUP1* array in (G). Cells were subjected to fluorescence microscopy on days 0, 3, and 10. Red, miRFP682; green, mNeonGreen; blue, mTagBFP; magenta, mCherry; grey, DIC. Scale bar, 20 µm.

(J) Alteration of CNA/G during BITREx. CNA/G was calculated for the periods from day 0 to 3, day 3 to 10, and day 0 to 10. *P < 0.05 (Student's t-test). The CNA/G between days 3 and 10 is significantly lower than that between days 0 and 3 in the absence of *ARS305*, but not in its presence. This is presumably because the desirable replication fork directionality at the nick was similar between ARS-less and ARS-containing arrays while they remained relatively short but could not be maintained in the ARS-less arrays as they expanded, unlike in the ARS-containing arrays.

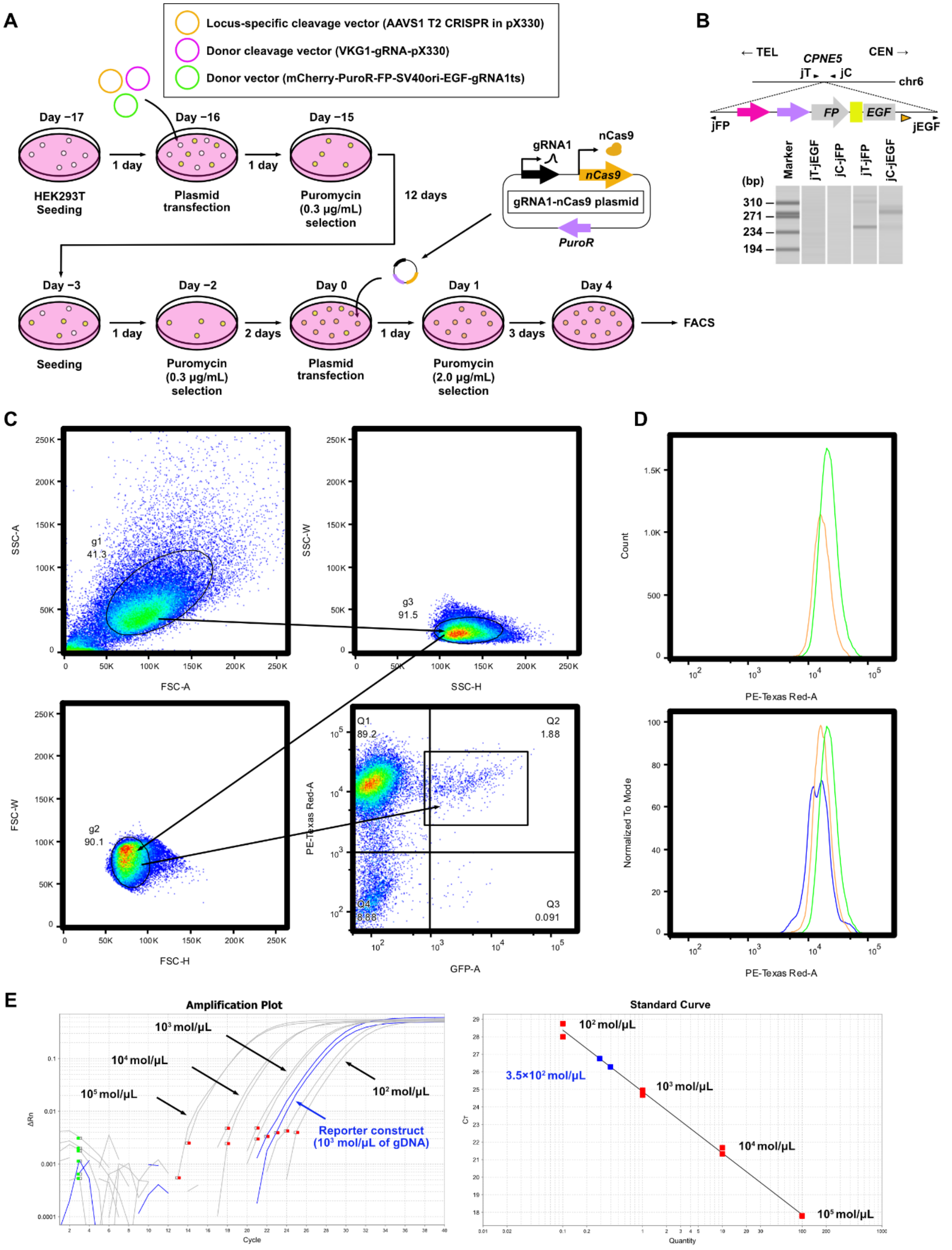

(legend on next page)

Figure S9: BITREx in mammalian cells, related to Figure 6

(A) Experimental procedure for BITREx in HEK293T cells. Using the three vectors for the VIKING method, we integrated the reporter construct into the *AAVS1* locus. Following the selection of HEK293T cells with the integrated construct by low concentration of puromycin, the gRNA1-nCas9 co-expression plasmid was transfected and selected by high concentration of puromycin. These cells were used for flow sorting.

(B) PCR genotyping of the *CPNE5* locus. PCR analysis using four primer combinations shown in the bottom panel consistently confirmed that the *EGFP* reconstitution reporter cassette was integrated as illustrated in the top panel.

(C) Light scatter-based gating of the cell population depicted in Figure 6C.

(D) Similar to Figure 6E, except fluorescence of mCherry was detected using the PE-Texas Red A channel. Based on these data and those in Figure 6E, we calculated the fluorescence ratio (EGFP/mCherry) in Figure 6F.

(E) Quantification of residual donor plasmid by qPCR. A primer pair spanning the cleavage site of the donor plasmid harboring the *EGFP* reconstitution reporter construct cassette, jEGF and jFP in (B), was designed to specifically detect residual donor plasmids while excluding signals from those integrated into the genome. qPCR analysis revealed that 350 moles of the residual donor plasmid were present in genomic DNA equivalent to 1,000 moles of a haploid genome. Considering the hypotriploid nature of HEK293 cells,[66] each puromycin-selected cell is estimated to contain ~1 copy of the residual plasmid.

Supplemental references

[S1] Gilbert, L.A., Larson, M.H., Morsut, L., Liu, Z., Brar, G.A., Torres, S.E., Stern-Ginossar, N., Brandman, O., Whitehead, E.V., Doudna, J.A. et al. (2013) CRISPR-mediated modular RNA-guided regulation of transcription in eukaryotes. Cell *154*, 442–451. https://doi.org/10.1016/j.cell.2013.06.044
